# Synthesis of spiro[dihydropyridine-oxindoles] via three-component reaction of arylamine, isatin and cyclopentane-1,3-dione

**DOI:** 10.3762/bjoc.9.2

**Published:** 2013-01-03

**Authors:** Yan Sun, Jing Sun, Chao-Guo Yan

**Affiliations:** 1 College of Chemistry & Chemical Engineering, Yangzhou University, Yangzhou 225002, China

**Keywords:** arylamine, cyclopentanedione, isatin, multicomponent reaction, spiro compound

## Abstract

A fast and convenient protocol for the synthesis of novel spiro[dihydropyridine-oxindole] derivatives in satisfactory yields was developed by the three-component reactions of arylamine, isatin and cyclopentane-1,3-dione in acetic acid at room temperature. On the other hand the condensation of isatin with two equivalents of cyclopentane-1,3-dione gave 3,3-bis(2-hydroxy-5-oxo-cyclopent-1-enyl)oxindole in high yields. The reaction mechanism and substrate scope of this novel reaction is briefly discussed.

## Introduction

The spirooxindole is among the most important class of naturally occurring substances, characterized by highly pronounced biological properties, and is also the core structure of many synthetic pharmaceuticals [1–2]. The various biological activities of spirooxindole derivatives have attracted much attention from organic chemists, and as a consequence, a number of methods have been reported for the preparation of spirooxindole-fused heterocycles [3–6]. Isatin and its derivatives may be the most useful starting materials or precursors in the synthesis of a wide number of spirocyclic oxindoles [7–8]. Due to its simple process, easy operation, efficiency and high atomic economy, the multicomponent reaction based on isatin and its derivatives have become an efficient method for the synthesis of various spirooxindoles in recent years [9–10]. It is known that the multicomponent reactions of isatins with in situ formed azomethine ylides have become the efficient synthetic procedure for constructing versatile spirooxindole systems [11–14]. Considering the above reports, and as part of our program aimed at developing new multicomponent reactions for the construction of complex heterocyclic compounds, we wish in this work to report the efficient synthesis of unprecedented cyclopentyl-fused spiro[dihydropyridine-oxindoles] by the three-component reaction of arylamine, isatin and cyclopentane-1,3-dione.

## Results and Discussion

**Scheme 1:**
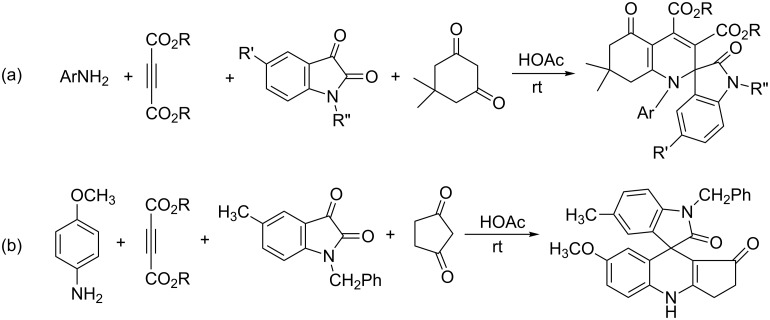
The four-component reactions containing dimedone (a) and cyclopentane-1,3-dione (b).

Recently we found that the four-component reactions of arylamine, acetylenedicarboxylate, isatin and dimedone in acetic acid resulted in the novel functionalized tetrahydrospiro[indoline-3,2’-quinoline] derivatives in moderate yields (Scheme 1a) [15]. In order to explore the generality of this four-component reaction, the reactivity of the other cyclic 1,3-diketones was also investigated. In an exploratory experiment, the four-component reaction of *p*-methoxyaniline, dimethyl acetylenedicarboxylate, 1-benzyl-5-methylisatin and cyclopentane-1,3-dione in acetic acid was carried out at room temperature. After workup, we were a little surprised to find that there was no unit of acetylenedicarboxylate in the obtained product (Scheme 1b). In another experiment, the three-component reaction of isatin, cyclopentane-1,3-dione and *β*-enamino ester, which was prepared in situ from the addition of *p*-methoxyaniline to dimethyl acetylenedicarboxylate in acetic acid according to our previously established procedure [15], also gave this new kind of spiro product in nearly the same yield. This result clearly indicated that the three-component reaction of arylamine, isatin and clopentane-1,3-dione gave the final spiro product, while the acetylenedicarboxylate could not take part in the reaction.

Attention was therefore turned to evaluate the generality of the three-component reaction of arylamine, isatin and cyclopentane-1,3-dione. Under similar conditions various arylamines and isatins with different substituents reacted with cyclopentane-1,3-dione in acetic acid at room temperature for 8–10 hours to afford the corresponding spiro[dihydropyridine-oxindole] compounds **1a**–**1k** in good yields. The results are shown in Table 1. From these results we could see that only anilines with electron-donating alkyl and alkoxy groups reacted smoothly. When anilines with electron-withdrawing *p*-chloro, *p*-bromo, *m*-nitro, or *p*-nitro groups were used in this three-component reaction, no expected spiro[dihydropyridine-oxindole] could be separated from the reaction system. On the other hand, the reaction of *α-*naphthylamine also gave good yields of spiro compound **1l** (Table 1, entry 12).

**Table 1:** Synthesis of spiro[dihydropyridine-oxindole] from three-component reactions.

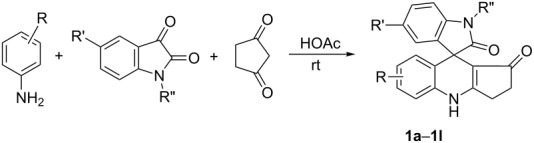					

Entry	Compound	R	R’	R”	Yield (%)

1	**1a**	*p-*CH_3_O	CH_3_	CH_2_Ph	88
2	**1b**	*p-*CH_3_	CH_3_	CH_2_Ph	85
3	**1c**	*p*-CH_3_CH_2_O	CH_3_	CH_2_Ph	60
4	**1d**	*p*-(CH_3_)_2_CH	CH_3_	CH_2_Ph	60
5	**1e**	*p*-(CH_3_)_3_C	CH_3_	CH_2_Ph	65
6	**1f**	*p-*CH_3_	H	CH_2_Ph	74
7	**1g**	*p-*CH_3_O	Cl	*n*-C_4_H_9_	52
8	**1h**	*p*-CH_3_	CH_3_	*n*-C_4_H_9_	82
9	**1i**	*p*-CH_3_	Cl	*n*-C_4_H_9_	56
10	**1j**	*p*-CH_3_	F	*n*-C_4_H_9_	58
11	**1k**	*p*-OH	CH_3_	*n*-C_4_H_9_	79
12	**1l**	α-naphthyl	CH_3_	CH_2_Ph	67

**Figure 1:**
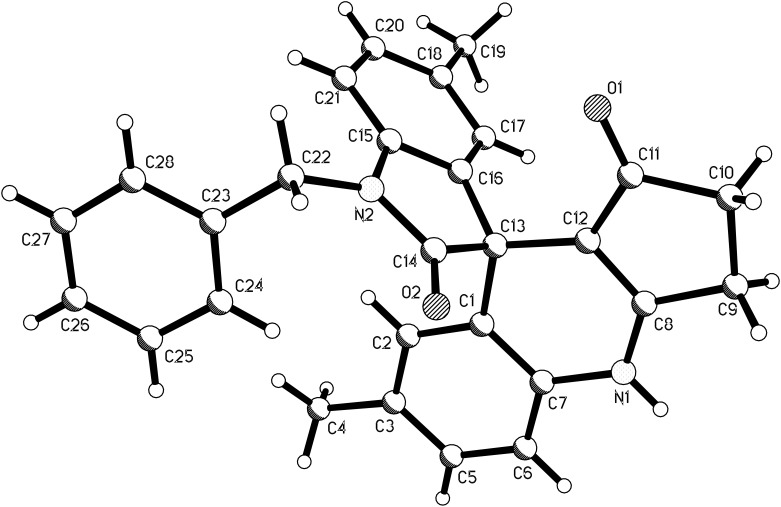
Molecular structure of spiro[dihydropyridine-oxindole] **1b**.

The structures of spiro compounds **1a**–**1l** were fully characterized by ^1^H and ^13^C NMR, HRMS, and IR spectra and were further confirmed by a single-crystal X-ray diffraction study performed for the compound **1b** (Figure 1).

**Figure 2:**
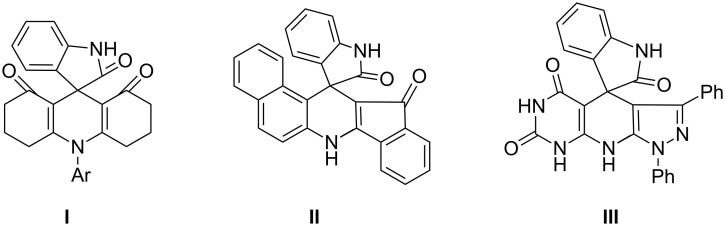
The two kinds of spiro compounds from reactions of isatins with arylamines and cyclic 1,3-diketones.

It should be pointed out that the structure of the obtained spiro compounds is very interesting, in which the oxindole was connected to the *ortho-*position of the amino group of arylamine. It is well known that the one-pot reactions of arylamine, isatin and cyclic 1,3-ketone under different catalytic conditions usually gave a kind of spiro[pyridine-oxindole] (**I** in Figure 2) as the main product, in which the arylamine only provided the amino group to form the pyridyl ring [16–21]. There are only very few papers describing that either 2-naphthylamine [22–25], functionalized 5-aminopyrazoles [26–28], or 2-aminobenzothiazoles [29] reacted with isatin and cyclic 1,3-dicarbonyl compounds to give the similar spiro[dihydropyridine-oxindole] (**II**, **III** in Figure 2), in which both the amino group and the aryl ring were involved in the construction of the pyridyl ring. In these cases only some special amines such as naphthylamine or heterocyclic amines were employed. It is well known that the reactivity at the α-position of 2-naphthylamine and the heterocyclic amine is much higher than that at the *ortho*-position of aniline. To the best of our knowledge, this new reaction provided the first example of normally substituted aniline showing this kind of reaction pattern.

**Figure 3:**
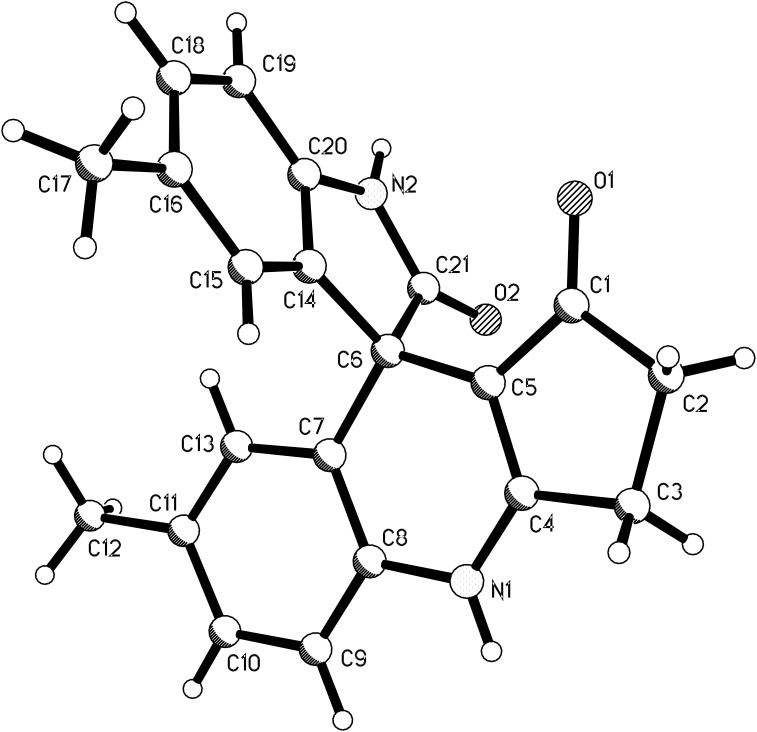
Molecular structure of spiro[dihydropyridine-oxindole] **2f**.

Encouraged by this success, we extended this three-component reaction to other isatins. When isatins without *N*-substituent were used under similar conditions, the reaction successfully resulted in the expected spiro[dihydropyridine-oxindole] **2a**–**2g** in lower yields (Table 2) and byproducts **3a**–**3d**, which obviously came from the condensation of isatins with two molar cyclopentane-1,3-diones. It is mentioned in Table 1 that isatins with an *N*-substituent afforded solely spiro[dihydropyridine-oxindole] **1a**–**1l** in satisfactory yields. Trying to increase the yields of spiro compounds and decrease the yields of condensation products was not successful. Both the spiro compounds **2a**–**2g** and condensation products **3a**–**3d** have low solubility in common organic solvents, such as chloroform, ethanol and THF, and could be partially dissolved in DMF. The structures of them were successfully established by spectroscopic methods and single crystal determination of compound **2f** (Figure 3).

**Table 2:** Synthesis of spiro[dihydropyridine-oxindole] from three-component reactions.

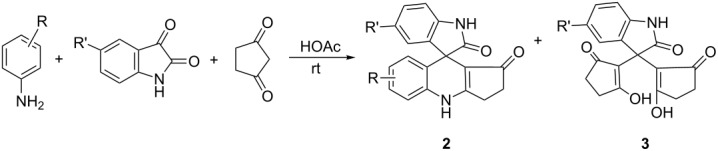						

Entry	R	R’	Compound **2**	Yield (%)	Compound **3**	Yield (%)

1	*p*-CH_3_O	H	**2a**	35	**3a**	20
2	*p*-CH_3_O	CH_3_	**2b**	36	**3b**	18
3	*p*-CH_3_O	Cl	**2c**	35	**3c**	16
4	*p*-CH_3_O	F	**2d**	30	**3d**	23
5	*p*-CH_3_	H	**2e**	26	**3a**	25
6	*p*-CH_3_	CH_3_	**2f**	35	**3b**	15
7	*p*-CH_3_	Cl	**2g**	32	**3c**	20

**Scheme 2:**
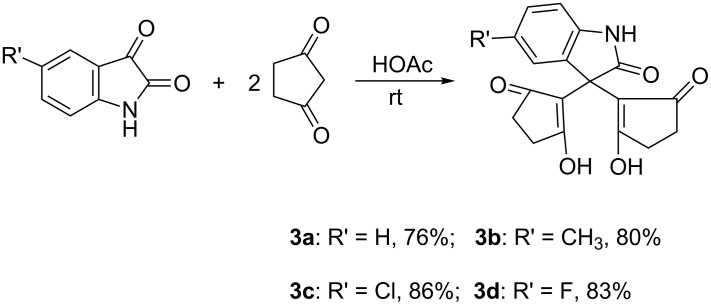
Condensation reactions of isatins with cyclopentane-1,3-dione.

**Figure 4:**
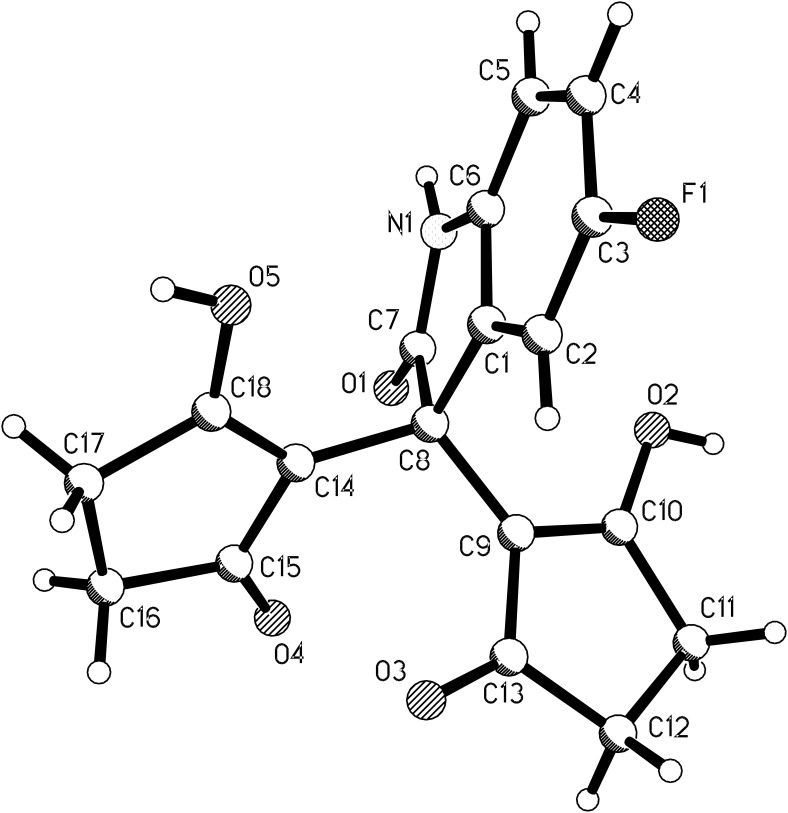
Molecular structure of compound **3d**.

A literature survey showed that even though there are a lot of reports about the condensation of isatins with cyclic 1,3-dicarbonyl compounds [30–33], the condensation reaction of isatin with cyclopentane-1,3-dione seemed still not to have been investigated and the 3,3-bis(2-hydroxy-5-oxo-cyclopent-1-enyl)oxindoles **3a**–**3d** have not been prepared until now. Thus, the direct condensation of isatins with two molar cyclopentane-1,3-dione was carried out in acetic acid and the desired condensation products **3a**–**3d** were obtained in high yields (Scheme 2). Then full characterization data were provided for them, and the single-crystal structure of compound **3d** was determined (Figure 4). ^1^H NMR spectra of 3,3-bis(2-hydroxy-5-oxo-cyclopent-1-enyl)oxindoles **3a**–**3d** showed a broad signal of two hydroxy groups at about 11.55 ppm, which clearly indicates cyclopentane-1,3-dione units exist in enol form. In the crystal structure of **3d** it is clearly seen that there is one carbonyl group and one enol group in each cyclopentane-1,3-dione moiety.

**Scheme 3:**
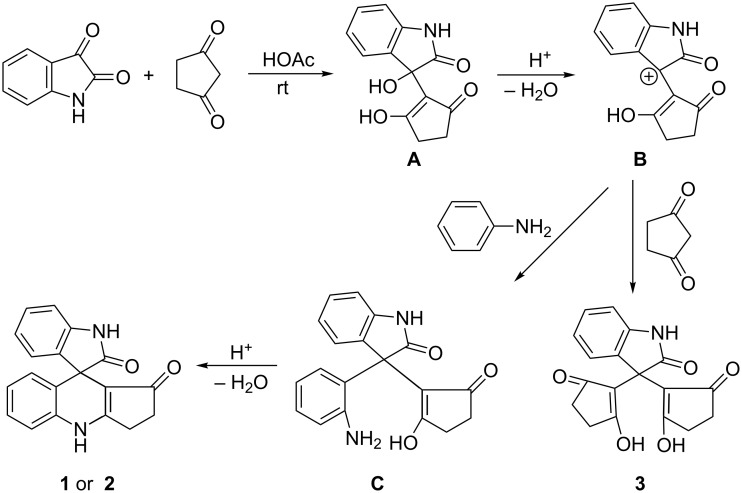
Proposed reaction mechanism for the three-component reaction.

Although at present the exact mechanism of this three-component reaction is not very clear, a plausible reaction mechanism for the formation of spiro[dihydropyridine-oxindoles] is presented based on the similar multicomponent reactions of isatins [22–29]. Firstly, the reaction of isatin with one equivalent of cyclopentane-1,3-dione in acetic acid forms an aldol adduct (**A** in Scheme 3). Secondly, a carbonium ion intermediate **B** was formed by acidic dehydration of adduct **A**. Then further reaction of carbonium ion **B** with a second equivalent of cyclopentane-1,3-dione gave the double condensation product **3**. On the other hand the carbonyl ion **B** reacted with the arylamine to give intermediate **C**. Then the intramolecular dehydration of **C** resulted in the final spiro compound **1** or **2**. In this reaction process the reactivity of arylamine played an important factor. The inactive arylamine bearing electron-withdrawing groups could not react with carbonium ion **B**, thus could not give the expected spiro compound.

## Conclusion

In conclusion, we have described a one-pot three-component reaction of arylamine, isatin and cyclopentane-1,3-dione and found an efficient procedure for the synthesis of a new type of polysubstituted spiro[dihydropyridine-oxindoles]. The reaction mechanism and substrate scope of this novel reaction were briefly discussed. Prominent among the advantages of this new method are operational simplicity, good yields of products in short reaction times, and easy workup procedures. Further expansion of the reaction scope and synthetic applications of this methodology are in progress in our laboratory.

## Experimental

**Reagents and apparatus:** All reagents and solvents were commercially available with analytical grade and used as received. Evaporative removal of organic solvents was carried out with a rotary evaporator in conjunction with a water jet pump. Melting points were taken on a hot-plate microscope apparatus and were uncorrected. ^1^H and ^13^C NMR spectra were recorded on a Bruker AV-600 instrument. IR spectra were obtained on a Bruker Tensor27 spectrometer (KBr disc). HRMS were measured on an AB 5800 MALDI–TOF/TOF instrument. X-ray data were collected on a Bruker Smart APEX-2 diffractometer.

**Typical procedure for the synthesis of spiro[dihydropyridine-oxindoles] 1a–1l from the three-component reaction of arylamine, cyclopentane-1,3-dione and isatin:** A mixture of arylamine (2.0 mmol), isatin (2.0 mmol) and cyclopentane-1,3-dione (2.0 mmol, 0.196 g) in 10.0 mL acetic acid was stirred at room temperature for about 9–12 hours. The resulting precipitates were collected by filtration and washed with cold ethanol to give pure product for analysis. **1a**: white solid, 88%; mp >300 °C; ^1^H NMR (600 MHz, DMSO-*d*_6_) δ 10.29 (s, 1H, NH), 7.51 (d, *J* = 7.8 Hz, 2H, ArH), 7.33 (t, *J* = 7.8 Hz, 2H, ArH), 7.27 (d, *J* = 7.2 Hz, 1H, ArH), 7.00 (d, *J* = 8.4 Hz, 1H, ArH), 6.94 (d, *J* = 7.8 Hz, 1H, ArH), 6.84–6.82 (m, 1H, ArH), 6.75 (d, *J* = 7.8 Hz, 1H, ArH), 6.70 (brs, 1H, ArH), 5.90 (d, *J* = 8.4 Hz, 1H, ArH), 4.99–4.91 (m, 2H, CH_2_), 3.47 (s, 3H, OCH_3_), 2.78–2.77 (m, 2H, CH_2_), 2.26–2.25 (m, 2H, CH_2_), 2.13 (s, 3H, CH_3_); ^13^C NMR (150 MHz, DMSO-*d*_6_) δ 198.3, 177.6, 165.9, 155.4, 139.5, 137.1, 136.6, 131.6, 130.3, 128.4, 128.1, 127.3, 127.2, 124.5, 124.4, 117.6, 113.9, 112.5, 108.6, 108.0, 56.0, 55.1, 50.5, 43.1, 32.8, 24.3, 20.5, 18.5; IR (KBr) ν: 3237, 3112, 3060, 2917, 1686, 1603, 1540, 1494, 1378, 1336, 1288, 1229, 1161, 1114, 1037, 859, 818 cm^−1^; HRMS–ESI (*m*/*z*): [M − H]^−^ calcd. for C_28_H_23_N_2_O_3_: 435.1714; found, 435.1711.

**Typical procedure for the synthesis of spiro[dihydropyridine-oxindoles] 2a–2g from three-component reaction of arylamine, cyclopentane-1,3-dione and isatin:** A mixture of arylamine (2.0 mmol), isatin (2.0 mmol) and cyclopentane-1,3-dione (2.0 mmol, 0.196 g) in 10.0 mL acetic acid was stirred at room temperature for about 9–12 hours. The resulting precipitates were collected by filtration and washed with cold ethanol to give the product. Recrystallization in DMF resulted in pure product for analysis.

**2a**: white solid, 35%; mp >300 °C; ^1^H NMR (600 MHz, DMSO-*d*_6_) δ 10.41 (s, 1H, NH), 10.21 (s, 1H, NH), 7.15–7.13 (m, 1H, ArH), 6.97 (d, *J* = 8.4 Hz, 1H, ArH), 6.87 (d, *J* = 7.8 Hz, 1H, ArH), 6.84–6.82 (m, 2H, ArH), 6.80 (d, *J* = 6.6 Hz, 1H, ArH), 6.61 (s, 1H, ArH), 5.99 (d, *J* = 3.0 Hz, 1H, ArH), 3.54 (s, 3H, OCH_3_), 2.74–2.72 (m, 2H, CH_2_), 2.22–2.20 (m, 2H, CH_2_); ^13^C NMR (150 MHz, DMSO-*d*_6_) δ 198.2, 179.2, 165.7, 155.3, 141.4, 137.9, 130.3, 127.7, 124.6, 123.9, 121.8, 117.4, 113.3, 112.9, 109.2, 108.1, 55.2, 50.9, 32.8, 24.2; IR (KBr) ν: 3203, 3108, 2966, 2925, 2833, 1703, 1661, 1590, 1538, 1494, 1388, 1330, 1288, 1251, 1194, 1162, 1124, 1039, 911, 857, 805 cm^−1^; HRMS–ESI (*m*/*z*): [M − H]^−^ calcd. for C_20_H_15_N_2_O_3_: 331.1088; found, 331.1088.

**Typical procedure for the synthesis of condensation products 3a–3c from condensation reaction of cyclopentane-1,3-dione and isatin:** A mixture of isatin (2.0 mmol) and cyclopentane-1,3-dione (2.0 mmol, 0.196 g) in 10.0 mL acetic acid was stirred at room temperature overnight. Then, a second portion of cyclopentane-1,3-dione (2.0 mmol, 0.196 g) was added to the system, and the solution was stirred at room temperature for 12 hours. The resulting precipitates were collected by filtration and washed with cold ethanol to give the pure product for analysis.

**3a**: white solid, 76%; mp 239–241 °C; ^1^H NMR (600 MHz, DMSO-*d*_6_) δ 11.57 (brs, 2H, OH), 10.42 (s, 1H, NH), 7.23 (d, *J* = 6.6 Hz, 1H, ArH), 7.02 (brs, 1H, ArH), 6.75 (brs, 1H, ArH), 6.68 (d, *J* = 7.2 Hz, 1H, ArH), 2.28 (brs, 8H, CH_2_); ^13^C NMR (150 MHz, DMSO-*d*_6_) δ 179.0, 141.6, 132.7, 132.6, 126.8, 126.7, 125.7, 120.6, 112.4, 108.5, 43.3, 30.4, 30.3, 30.2, 30.1, 30.0, 29.9, 29.8, 29.7; IR (KBr) ν: 3196, 2925, 1676, 1612, 1573, 1478, 1435, 1385, 1362, 1306, 1232, 1110, 1069, 1040, 881 cm^−1^; HRMS–ESI (*m*/*z*): [M − H]^−^ calcd. for C_18_H_14_NO_5_: 324.0877; found, 324.0874.

Crystallographic data (CIF) of all new compounds are available free of charge via the Internet. Single crystal data for compound **1b** (CCDC 869516), **2f** (CCDC 869519) and **3d** (CCDC 881763) have been deposited in the Cambridge Crystallographic Data Centre. These data can be obtained free of charge via http://www.ccdc.cam.ac.uk./data_request/cif

## Supporting Information

File 1 Spectroscopic and analytical data.
