# Supplementary material for: Synthesis of spiro[dihydropyridine-oxindoles] via three-component reaction of arylamine, isatin and cyclopentane-1,3-dione
Source: Beilstein J Org Chem. 2013 Jan 3;9:8–14. doi: 10.3762/bjoc.9.2 (PMC3566796; doi:10.3762/bjoc.9.2)

Supporting Information

for

Synthesis of spiro[dihydropyridine-oxindoles] via three-component reaction of arylamine, isatin and cyclopentane-1,3-dione

Yan Sun, Jing Sun, Chao-Guo Yan*

College of Chemistry & Chemical Engineering, Yangzhou University,

Yangzhou 225002, China

Email: Chao-Guo Yan - cgyan@yzu.edu.cn

* Corresponding author

Spectroscopic and analytical data

1a: white solid, 88%, mp >300 °C; ^{1}H NMR (600 MHz, DMSO-d_6) δ: 10.29 (s, 1H, NH), 7.51 (d, J = 7.8 Hz, 2H, ArH), 7.33 (t, J = 7.8 Hz, 2H, ArH), 7.27 (d, J = 7.2 Hz, 1H, ArH), 7.00 (d, J = 8.4 Hz, 1H, ArH), 6.94 (d, J = 7.8 Hz, 1H, ArH), 6.84–6.82 (m, 1H, ArH), 6.75 (d, J = 7.8 Hz, 1H, ArH), 6.70 (brs, 1H, ArH), 5.90 (d, J = 8.4 Hz, 1H, ArH), 4.99–4.91 (m, 2H, CH_2), 3.47 (s, 3H, OCH_3), 2.78–2.77 (m, 2H, CH_2), 2.26–2.25 (m, 2H, CH_2), 2.13 (s, 3H, CH_3); ^{13}C NMR (150 MHz, DMSO-d_6) δ: 198.3, 177.6, 165.9, 155.4, 139.5, 137.1, 136.6, 131.6, 130.3, 128.4, 128.1, 127.3, 127.2, 124.5, 124.4, 117.6, 113.9, 112.5, 108.6, 108.0, 56.0, 55.1, 50.5, 43.1, 32.8, 24.3, 20.5, 18.5; IR (KBr) υ: 3237, 3112, 3060, 2917, 1686, 1603, 1540, 1494, 1378, 1336, 1288, 1229, 1161, 1114, 1037, 859, 818 cm^{-1}; MS (m/z): HRMS (ESI) Calcd. for $C_{28}H_{23}N_2O_3$ ([M-H]$^-$): 435.1714. Found: 435.1711.

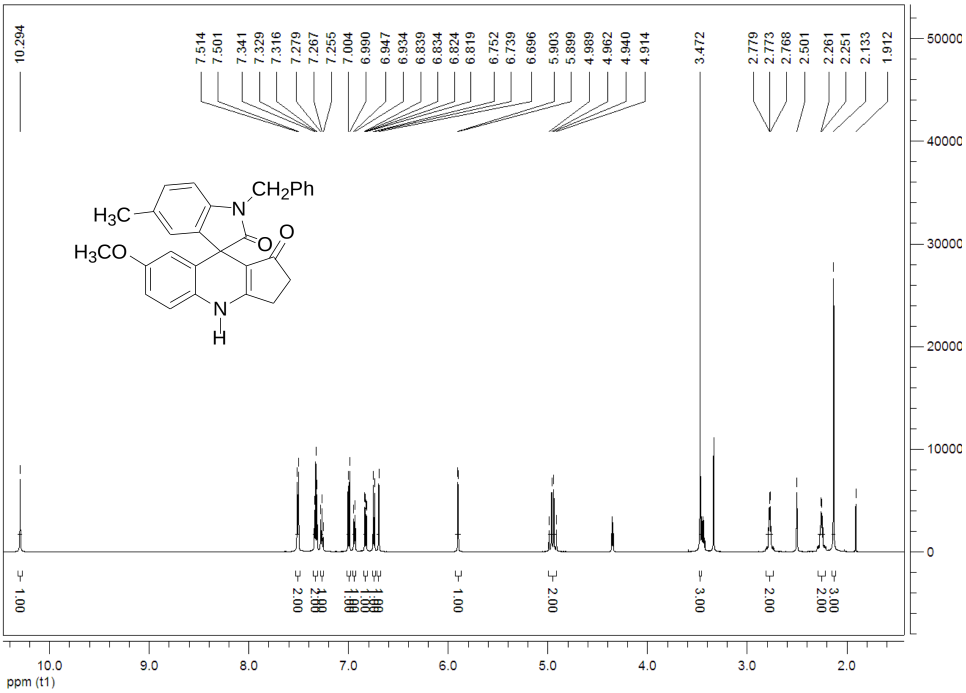

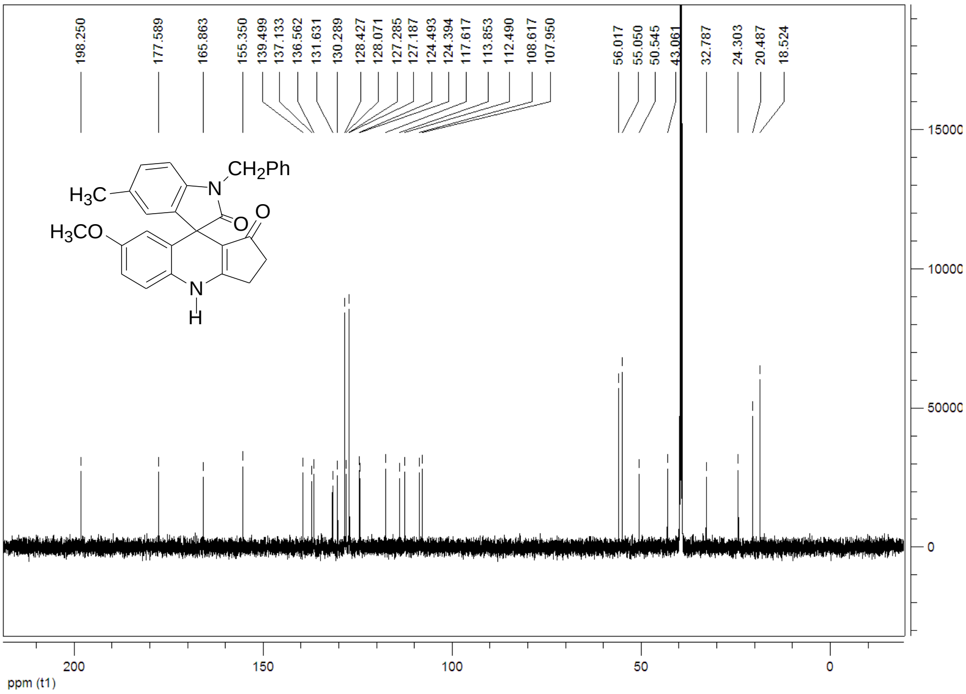

1b: white solid, 85%, mp 302–304 °C; ^{1}H NMR (600 MHz, DMSO-d_6) δ: 10.32 (s, 1H, NH), 7.51 (d, J = 7.8 Hz, 2H, ArH), 7.35 (t, J = 7.8 Hz, 2H, ArH), 7.28 (d, J = 7.2 Hz, 1H, ArH), 6.99 (d, J = 7.8 Hz, 1H, ArH), 6.93 (d, J = 7.2 Hz, 2H, ArH), 6.76 (d, J = 8.4 Hz, 1H, ArH), 6.68 (s, 1H, ArH), 6.17 (s, 1H, ArH), 4.98–4.92 (m, 2H, CH_2), 2.89–2.74 (m, 2H, CH_2), 2.27–2.24 (m, 2H, CH_2), 2.13 (s, 3H, CH_3), 2.00 (s, 3H, CH_3); ^{13}C NMR (150 MHz, DMSO-d_6) δ: 198.4, 177.8, 166.1, 139.5, 137.4, 136.6, 134.2, 132.3, 131.6, 129.0, 128.4, 127.5, 127.4, 127.2, 124.5, 123.3, 116.6, 108.9, 108.6, 50.2, 43.0, 32.8, 24.3, 20.5, 20.2; IR (KBr) υ: 3243, 3174, 3105, 3058, 2976, 2921, 1684, 1599, 1535, 1495, 1442, 1379, 1336, 1295, 1247, 1188, 1117, 1085, 1046, 946, 856, 819 cm^{-1}; MS (m/z): HRMS (ESI) Calcd. for $C_{28}H_{23}N_2O_2$ ([M-H]$^-$): 419.1765. Found: 419.1763.

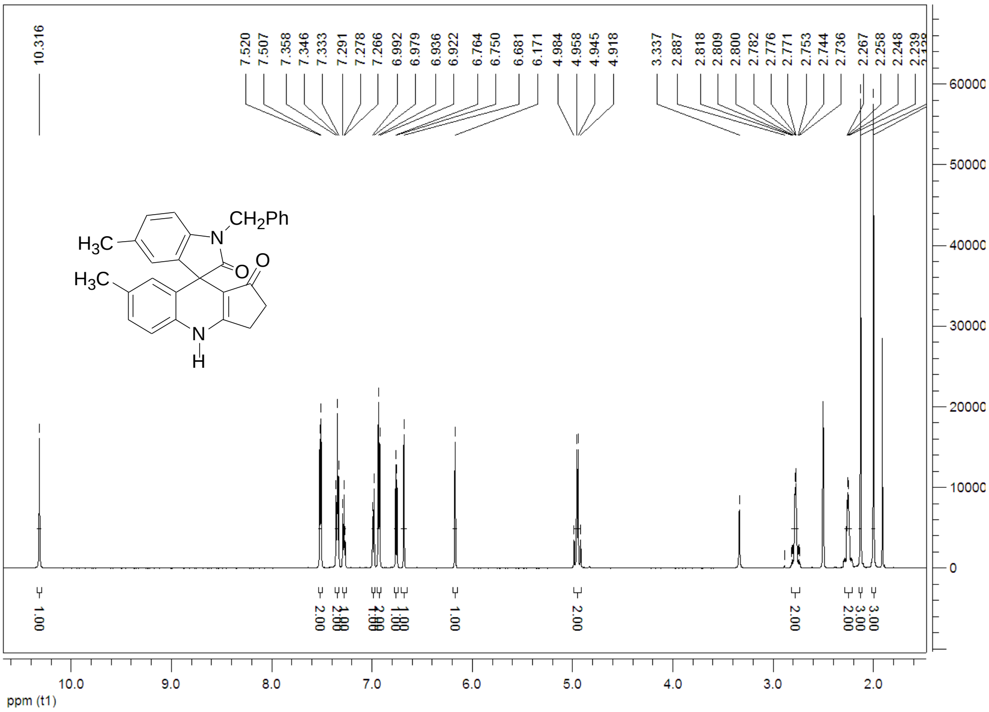

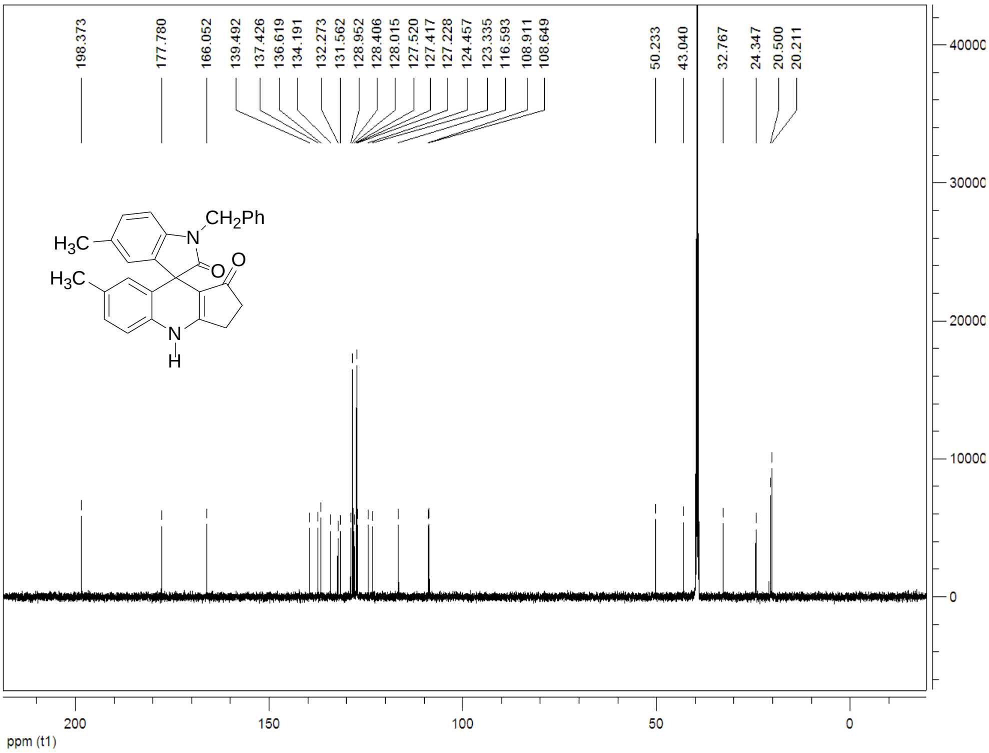

1c: white solid, 60%, mp 256–258 °C; ^{1}H NMR (600 MHz, DMSO-d_6) δ: 10.29 (s, 1H, NH), 7.51 (d, J = 7.2 Hz, 2H, ArH), 7.33 (t, J = 7.2 Hz, 2H, ArH), 7.27 (t, J = 7.2 Hz, 1H, ArH), 6.98 (d, J = 8.4 Hz, 1H, ArH), 6.94 (d, J = 8.4 Hz, 1H, ArH), 6.80 (dd, J^1 = 9.0 Hz, J^2 = 2.4 Hz, 1H, ArH), 6.76 (d, J = 8.4 Hz, 1H, ArH), 6.69 (s, 1H, ArH), 5.87 (d, J = 2.4 Hz, 1H, ArH), 4.99–4.92 (m, 2H, CH_2), 3.66 (q, J = 6.6 Hz, 2H, CH_2), 2.78–2.77 (m, 2H, CH_2), 2.27–2.24 (m, 2H, CH_2), 2.13 (s, 3H, CH_3), 1.16 (t, J = 6.6 Hz, 3H, CH_3); ^{13}C NMR (150 MHz, DMSO-d_6) δ: 198.3, 177.6, 165.9, 154.6, 139.5, 137.2, 136.6, 131.6, 130.1, 128.4, 128.0, 127.4, 127.2, 124.5, 124.4, 117.6, 114.3, 113.0, 108.6, 107.9, 63.0, 50.5, 43.0, 32.8, 24.3, 20.5, 14.4; IR (KBr) υ: 3233, 3180, 3111, 3051, 2973, 2925, 1678, 1622, 1600, 1539, 1497, 1476, 1382, 1335, 1247, 1230, 1183, 1159, 1106, 1084, 1042, 929, 895, 855, 825, 807 cm^{-1}; MS (m/z): HRMS (ESI) Calcd. for $C_{29}H_{25}N_2O_3$ ([M-H]$^-$): 449.1874. Found: 449.1873.

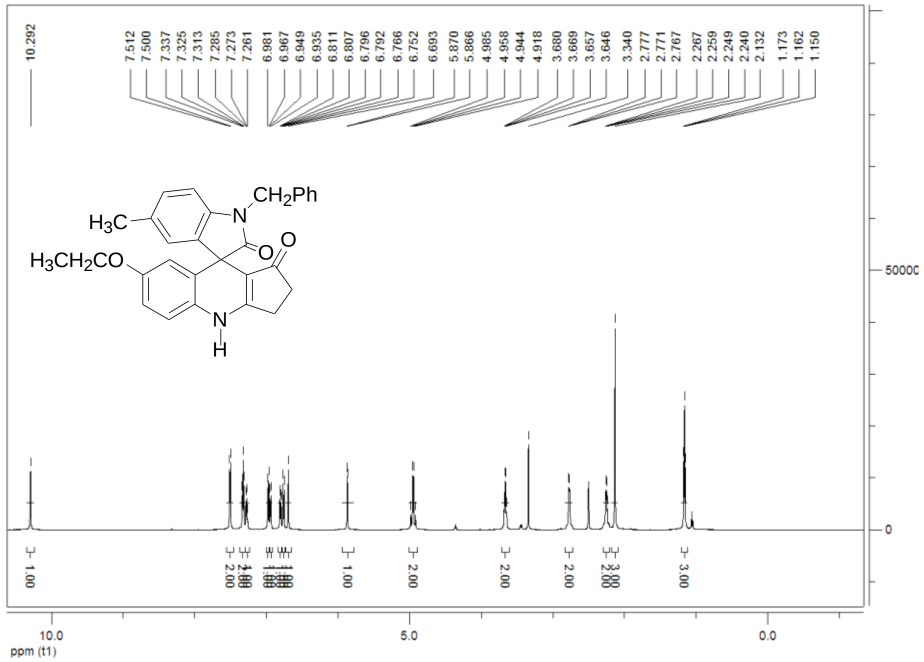

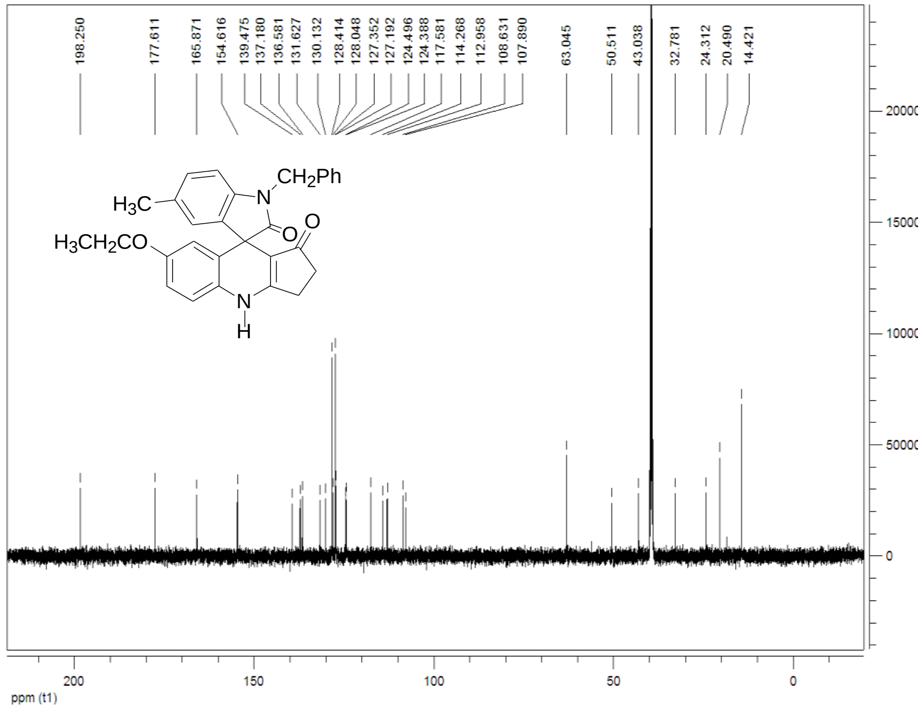

1d: white solid, 60%, mp 312–314 °C; ^{1}H NMR (600 MHz, DMSO-d_6) δ: 10.36 (s, 1H, NH), 7.50 (d, *J* = 7.8 Hz, 2H, ArH), 7.33 (t, *J* = 6.6 Hz, 2H, ArH), 7.28 (t, *J* = 7.2 Hz, 1H, ArH), 7.07 (d, *J* = 7.8 Hz, 1H, ArH), 6.97 (d, *J* = 7.8 Hz, 1H, ArH), 6.94 (d, *J* = 7.8 Hz, 1H, ArH), 6.79 (d, *J* = 7.8 Hz, 1H, ArH), 6.69 (s, 1H, ArH), 6.21 (s, 1H, ArH), 5.00 (d, *J* = 15.6 Hz, 1H, CH), 4.88 (d, *J* = 15.6 Hz, 1H, CH), 2.77–2.76 (m, 2H, CH_2), 2.54–2.53 (m, 1H, CH), 2.25–2.24 (m, 2H, CH_2), 2.13 (s, 3H, CH_3), 0.96 (d, *J* = 7.2 Hz, 3H, CH_3), 0.94 (d, *J* = 6.6 Hz, 3H, CH_3); ^{13}C NMR (150 MHz, DMSO-d_6) δ: 198.3, 177.8, 166.1, 143.3, 139.5, 137.4, 136.8, 134.6, 131.5, 128.4, 128.0, 127.4, 127.3, 126.0, 125.0, 124.4, ,123.2, 116.7, 108.9, 108.5, 50.3, 43.0, 32.8, 32.5, 24.3, 23.8, 23.5, 20.5; IR (KBr) υ: 3240, 3175, 3107, 3050, 2960, 2925, 1694, 1624, 1600, 1532, 1496, 1455, 1423, 1376, 1325, 1293, 1245, 1226, 1192, 1166, 1099, 1048, 966, 921, 880, 803 cm^{-1}; MS (*m*/*z*): HRMS (ESI) Calcd. for $C_{30}H_{27}N_2O_2$ ([M-H]$^-$): 447.2079. Found: 447.2073.

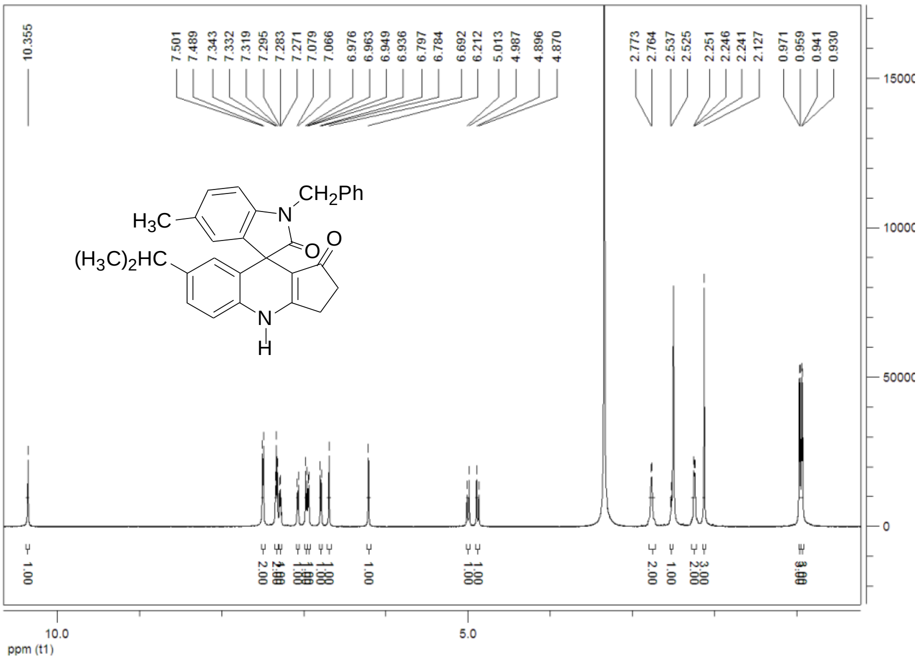

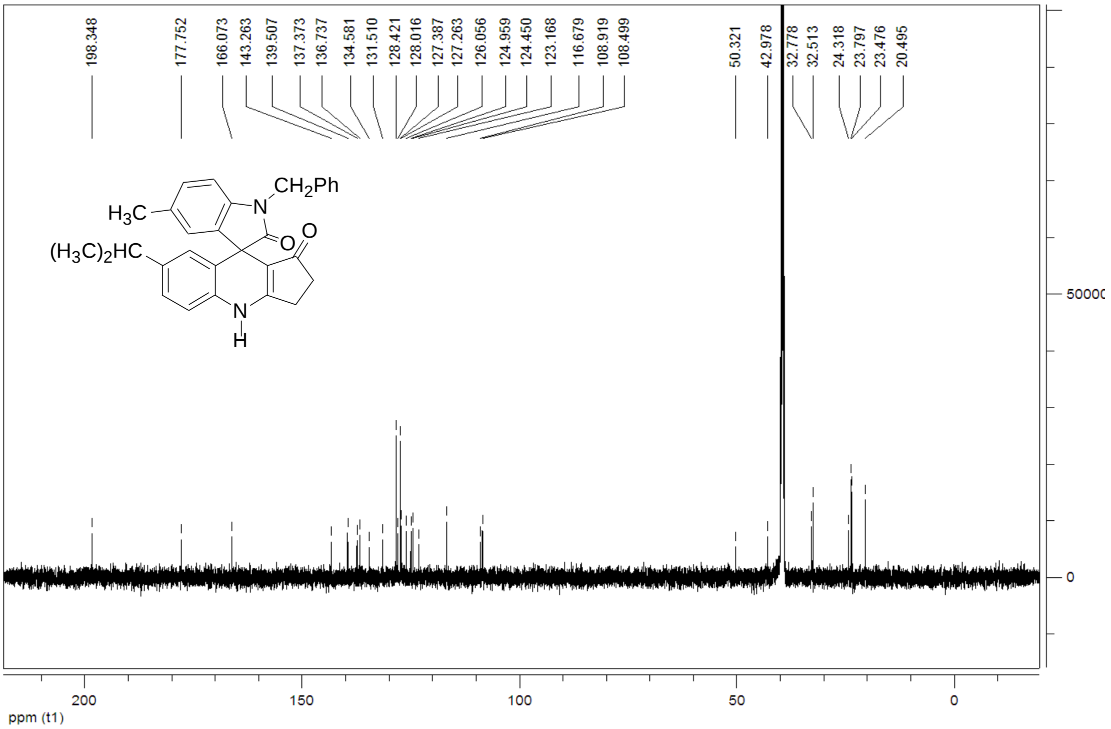

1e: white solid, 65%, mp >300 °C; ^{1}H NMR (600 MHz, DMSO-d_6) δ: 10.66 (s, 1H, NH), 7.49 (d, J = 7.2 Hz, 2H, ArH), 7.32 (t, J = 7.2 Hz, 2H, ArH), 7.28 (t, J = 6.6 Hz, 1H, ArH), 7.22 (d, J = 7.8 Hz, 1H, ArH), 7.03 (d, J = 7.8 Hz, 1H, ArH), 6.95 (d, J = 7.2 Hz, 1H, ArH), 6.79 (d, J = 7.8 Hz, 1H, ArH), 6.69 (s, 1H, ArH), 6.39 (s, 1H, ArH), 5.03 (d, J = 15.6 Hz, 1H, CH), 4.85 (d, J = 15.6 Hz, 1H, CH), 2.80–2.73 (m, 2H, CH_2), 2.24 (brs, 2H, CH_2), 2.13 (s, 3H, CH_3), 0.99 (s, 9H, CH_3); ^{13}C NMR (150 MHz, DMSO-d_6) δ: 198.3, 177.8, 166.1, 145.5, 139.5, 137.4, 136.7, 134.3, 131.5, 128.5, 128.0, 127.3, 127.2, 125.3, 124.4, 123.5, 122.8, 116.4, 108.4, 50.4, 43.0, 33.7, 32.8, 30.8, 24.3, 20.5; IR (KBr) υ: 3032, 2960, 2867, 1694, 1627, 1600, 1527, 1496, 1433, 1405, 1371, 1303, 1248, 1191, 1165, 1102, 1049, 840 cm^{-1}; MS (m/z): HRMS (ESI) Calcd. for $C_{31}H_{29}N_2O_2$ ([M-H]$^-$): 461.2207. Found: 461.2203.

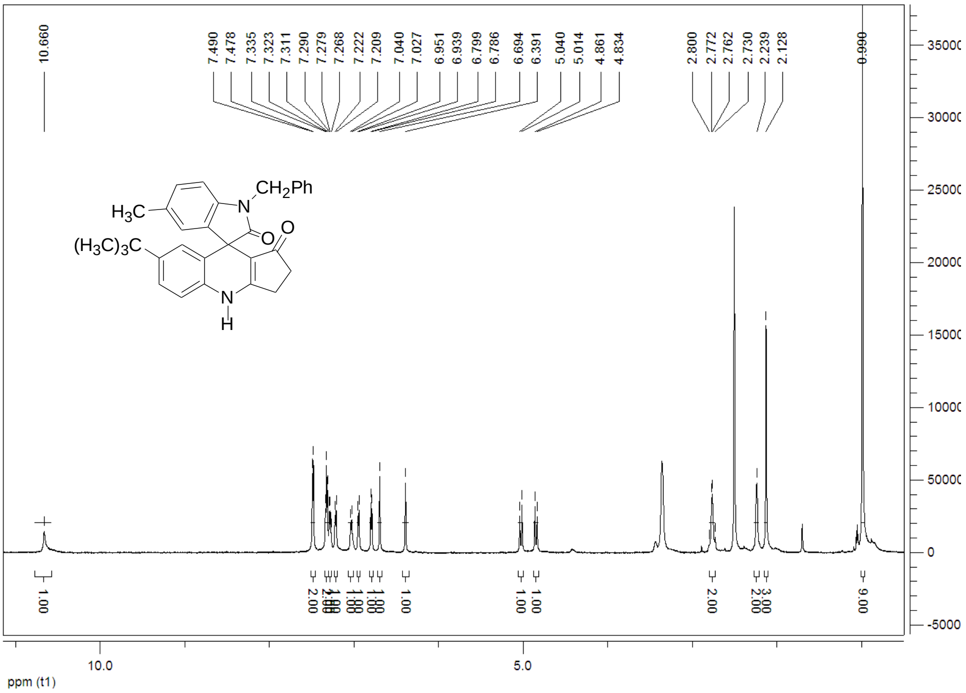

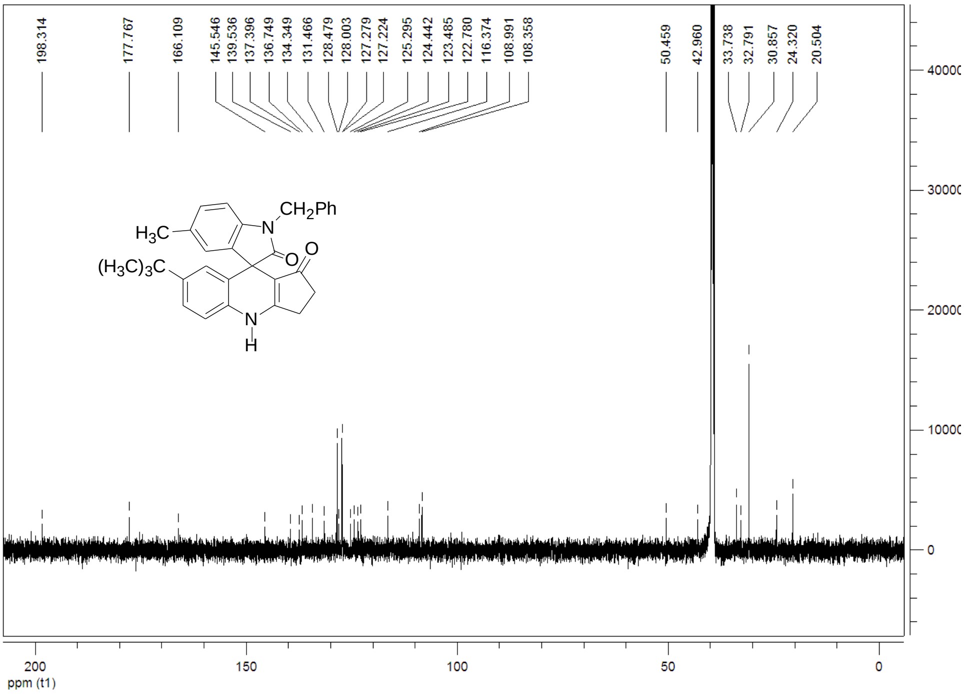

1f: white solid, 74%, mp 289–292 °C; ^{1}H NMR (600 MHz, DMSO-d_6) δ: 10.33 (s, 1H, NH), 7.52 (d, J = 7.8 Hz, 2H, ArH), 7.35 (t, J = 7.8 Hz, 2H, ArH), 7.28 (t, J = 7.2 Hz, 1H, ArH), 7.15–7.12 (m, 1H, ArH), 7.00–6.98 (m, 1H, ArH), 6.93 (d, J = 8.4 Hz, 1H, ArH), 6.90–6.86 (m, 3H, ArH), 6.17 (s, 1H, ArH), 5.01–4.95 (m, 2H, CH_2), 2.82–2.74 (m, 2H, CH_2), 2.29–2.21 (m, 2H, CH_2), 2.00 (s, 3H, CH_3); ^{13}C NMR (150 MHz, DMSO-d_6) δ: 198.3, 177.8, 166.1, 166.0, 141.8, 137.3, 136.6, 134.2, 129.0, 128.4, 127.7, 127.4, 127.3, 127.2, 123.9, 123.2, 122.6, 122.5, 116.6, 108.9, 108.8, 56.0, 50.2, 43.1, 43.0, 32.7, 24.3, 20.2, 18.5; IR (KBr) υ: 3245, 3178, 3106, 3056, 2918, 1680, 1602, 1531, 1494, 1374, 1337, 1300, 1246, 1217, 1173, 1087, 1047, 1005, 942, 889, 851, 817 cm^{-1}; MS (m/z): HRMS (ESI) Calcd. for $C_{27}H_{21}N_2O_2$ ([M-H]$^-$): 405.1609. Found: 405.1609.

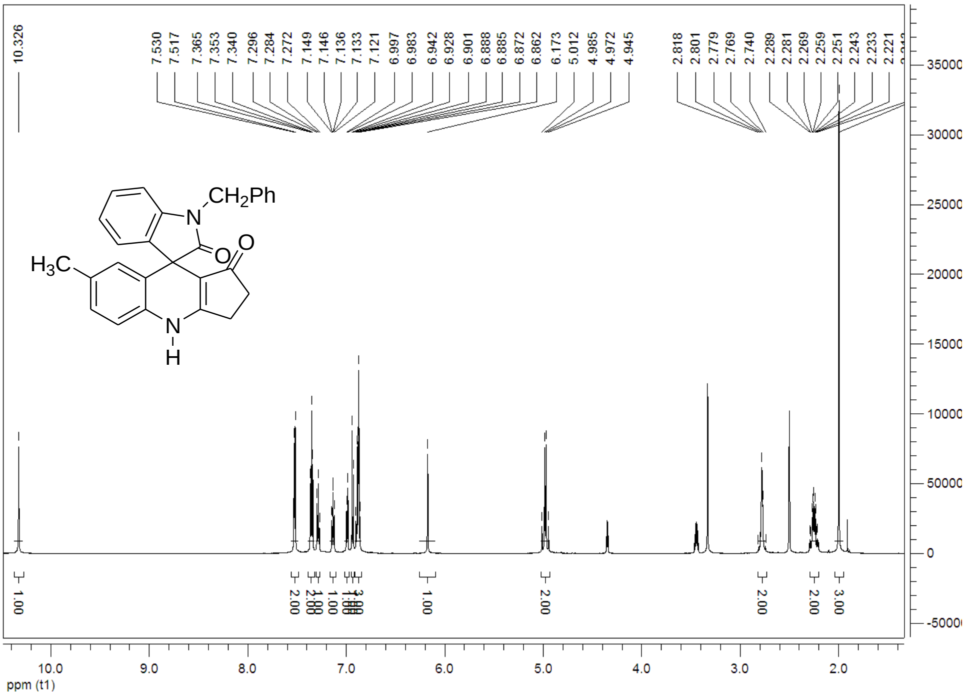

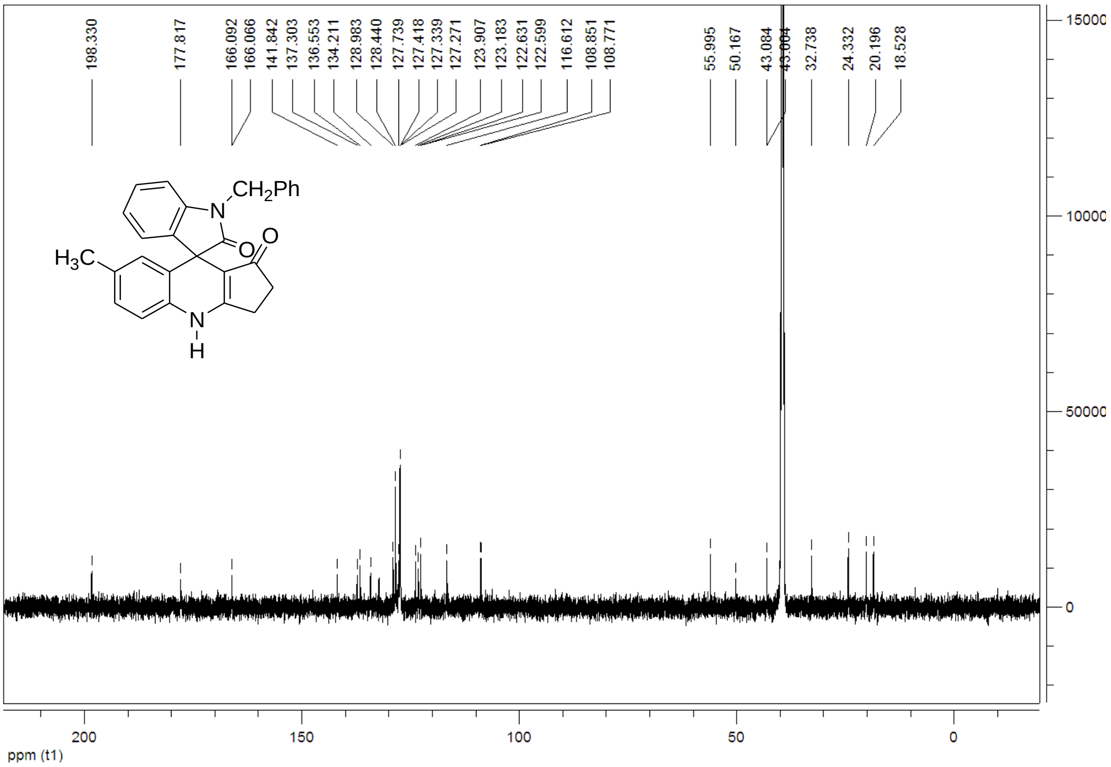

1g: white solid, 52%, mp 309–310 °C; ^{1}H NMR (600 MHz, DMSO-d_6) δ: 10.31 (s, 1H, NH), 7.31–7.29 (m, 1H, ArH), 7.13 (d, J = 8.4 Hz, 1H, ArH), 7.01 (d, J = 9.0 Hz, 1H, ArH), 6.88 (d, J = 2.4 Hz, 1H, ArH), 6.87–6.85 (m, 1H, ArH), 5.89 (d, J = 3.0 Hz, 1H, ArH), 3.77–3.67 (m, 2H, CH_2), 3.54 (s, 3H, OCH_3), 2.75–2.74 (m, 2H, CH_2), 2.23–2.22 (m, 2H, CH_2), 1.64–1.61 (m, 2H, CH_2), 1.39–1.36 (m, 2H, CH_2), 0.90 (t, J = 7.8 Hz, 3H, CH_3); ^{13}C NMR (150 MHz, DMSO-d_6) δ:198.2, 177.1, 165.9, 155.4, 141.4, 138.8, 130.3, 127.9, 126.3, 123.8, 123.5, 117.8, 114.0, 112.3, 109.9, 107.3, 56.0, 55.1, 50.5, 32.7, 29.1, 24.3, 19.5, 18.5, 13.7; IR (KBr) υ: 3236, 3183, 3111, 3049, 2954, 2868, 1685, 1624, 1603, 1540, 1494, 1433, 1379, 1344, 1249, 1191, 1160, 1105, 1040, 990, 910, 825 cm^{-1}; MS (m/z): HRMS (ESI) Calcd. for $C_{24}H_{22}ClN_2O_3$ ([M-H]$^-$): 421.1324. Found: 421.1322.

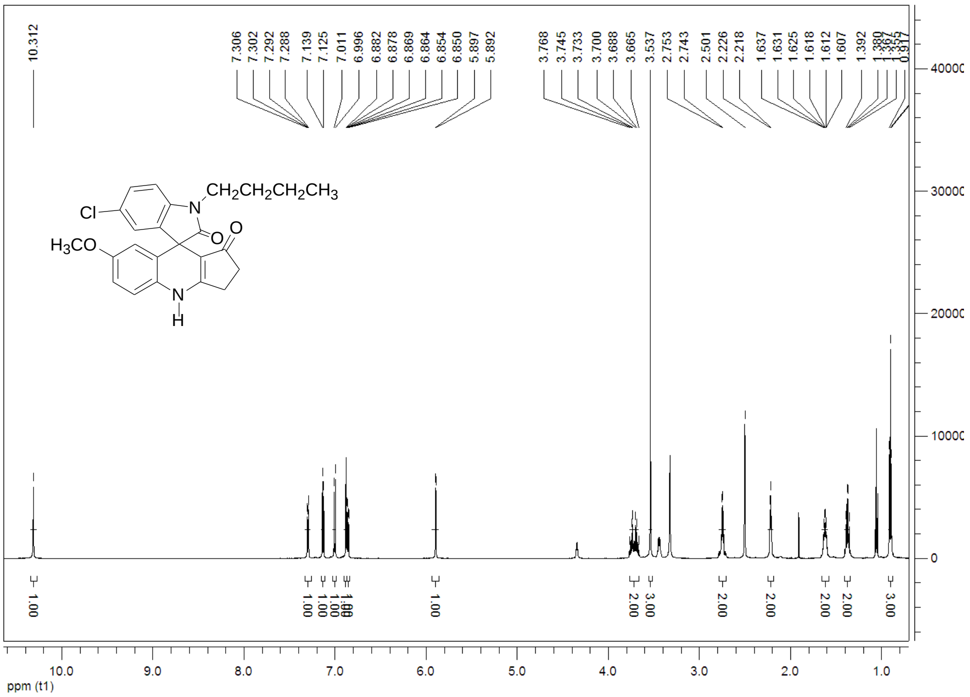

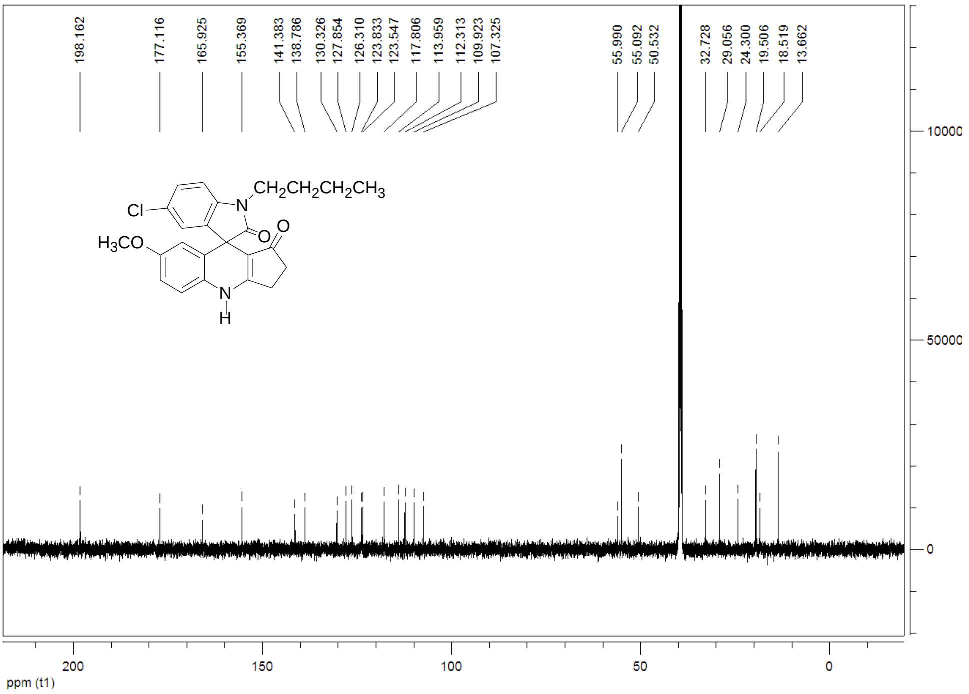

1h: white solid, 82%, mp 280–281 °C; ^{1}H NMR (600 MHz, DMSO-d_6) δ: 10.26 (s, 1H, NH), 7.02 (d, J = 7.8 Hz, 1H, ArH), 6.98 (d, J = 8.4 Hz, 1H, ArH), 6.95 (d, J = 7.8 Hz, 1H, ArH), 6.91 (d, J = 8.4 Hz, 1H, ArH), 6.66 (s, 1H, ArH), 6.20 (s, 1H, ArH), 3.72–3.66 (m, 2H, CH_2), 2.78–2.73 (m, 2H, CH_2), 2.21–2.19 (m, 2H, CH_2), 2.16 (s, 3H, CH_3), 2.03 (s, 3H, CH_3), 1.67–1.62 (m, 2H, CH_2), 1.42–1.36 (m, 2H, CH_2), 0.92 (t, J = 7.2 Hz, 3H, CH_3); ^{13}C NMR (150 MHz, DMSO-d_6) δ: 198.2, 177.5, 165.8, 140.1, 137.5, 134.3, 132.1, 131.1, 128.9, 128.1, 127.4, 124.5, 123.3, 116.5, 109.1, 108.0, 56.0, 50.2, 32.7, 29.1, 24.2, 20.5, 20.3, 19.6, 18.5, 13.7; IR (KBr) υ: 3212, 3170, 3045, 2952, 2919, 2862, 1682, 1633, 1604, 1530, 1494, 1441, 1376, 1297, 1247, 1195, 1163, 1125, 1105, 1045, 988, 912, 883, 856, 821 cm^{-1}; MS (m/z): HRMS (ESI) Calcd. for $C_{25}H_{25}N_2O_2$ ([M-H]$^-$): 385.1922. Found: 385.1920.

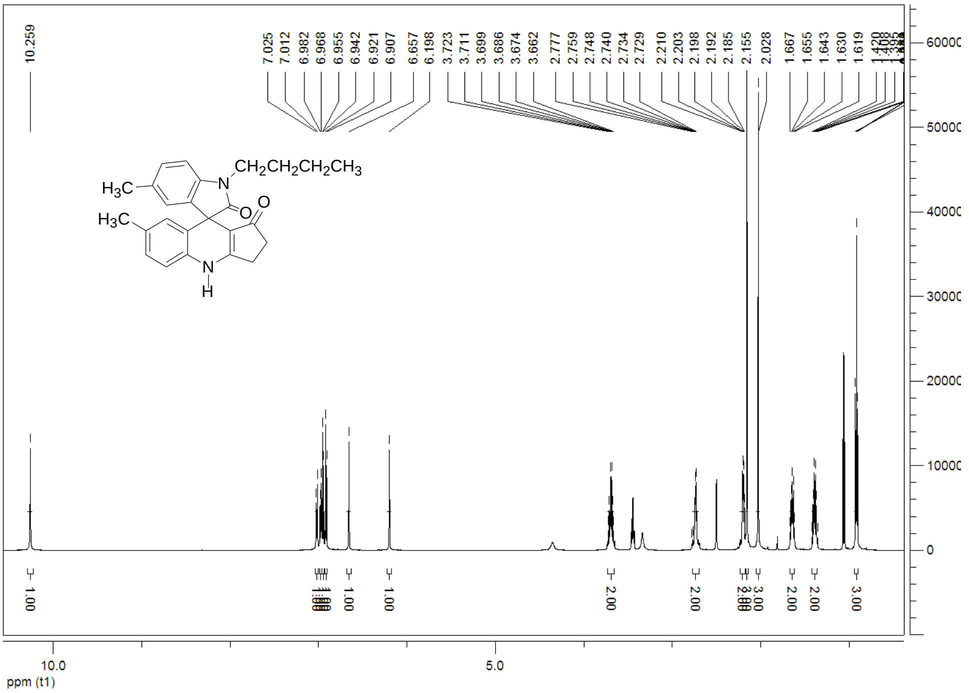

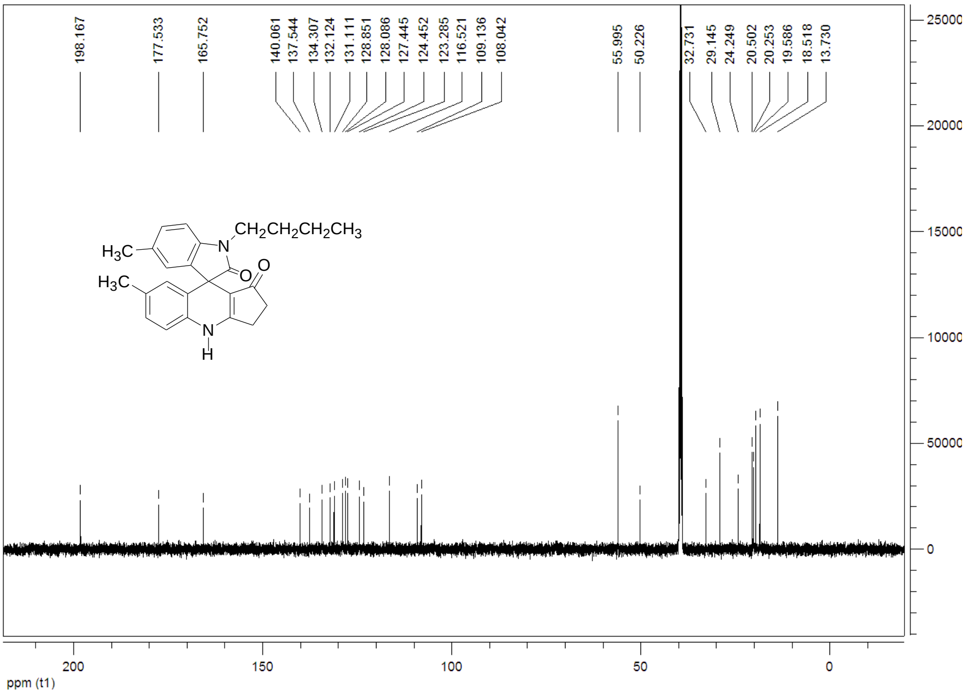

1i: white solid, 56%, mp 310–311 °C; ^{1}H NMR (600 MHz, DMSO-d_6) δ: 10.33 (s, 1H, NH), 7.29 (d, J = 8.4 Hz, 1H, ArH), 7.12 (d, J = 7.8 Hz, 1H, ArH), 7.01 (d, J = 7.8 Hz, 1H, ArH), 6.94 (d, J = 8.4 Hz, 1H, ArH), 6.87 (s, 1H, ArH), 6.21 (s, 1H, ArH), 3.73–3.71 (m, 2H, CH_2), 2.75–2.74 (m, 2H, CH_2), 2.22–2.21 (m, 2H, CH_2), 2.05 (s, 3H, CH_3), 1.64–1.62 (m, 2H, CH_2), 1.40–1.36 (m, 2H, CH_2), 0.91 (t, J = 7.2 Hz, 3H, CH_3); ^{13}C NMR (150 MHz, DMSO-d_6) δ: 198.3, 177.3, 166.1, 141.5, 139.1, 134.3, 132.4, 129.2, 127.8, 127.3, 126.2, 123.9, 122.3, 116.8, 109.9, 108.4, 56.0, 50.3, 32.7, 29.0, 24.3, 20.2, 19.5, 18.5, 13.7; IR (KBr) υ: 3216, 3173, 3098, 3036, 2952, 2923, 2865, 1686, 1632, 1603, 1530, 1493, 1429, 1378, 1345, 1300, 1248, 1187, 1109, 1150, 991, 909, 879, 829 cm^{-1}; MS (m/z): HRMS (ESI) Calcd. for $C_{24}H_{22}ClN_2O_2$ ($[M-H]^-$): 405.1375. Found: 405.1375.

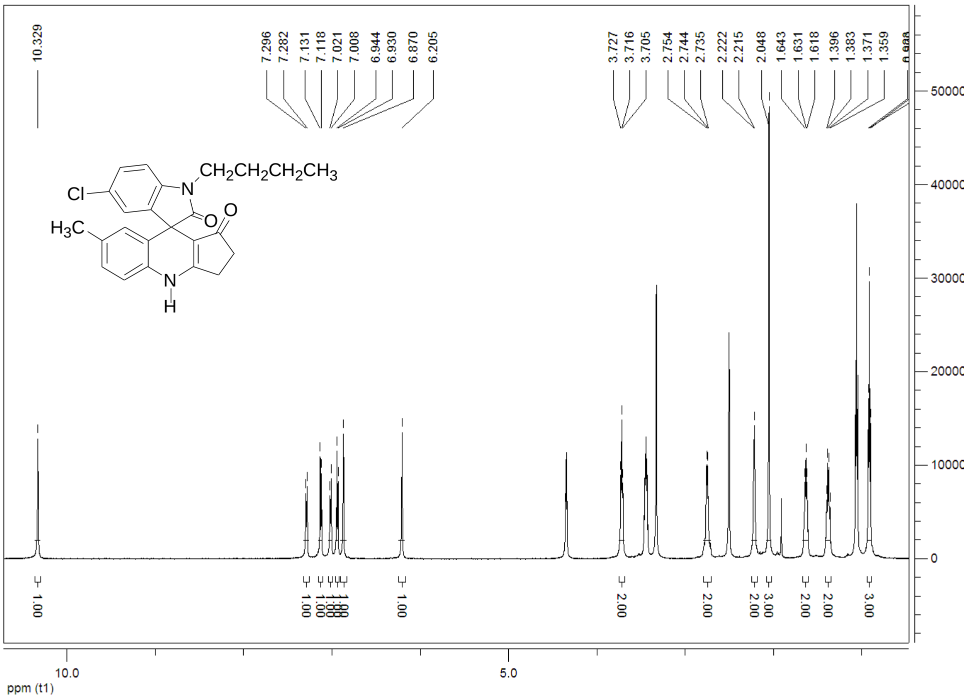

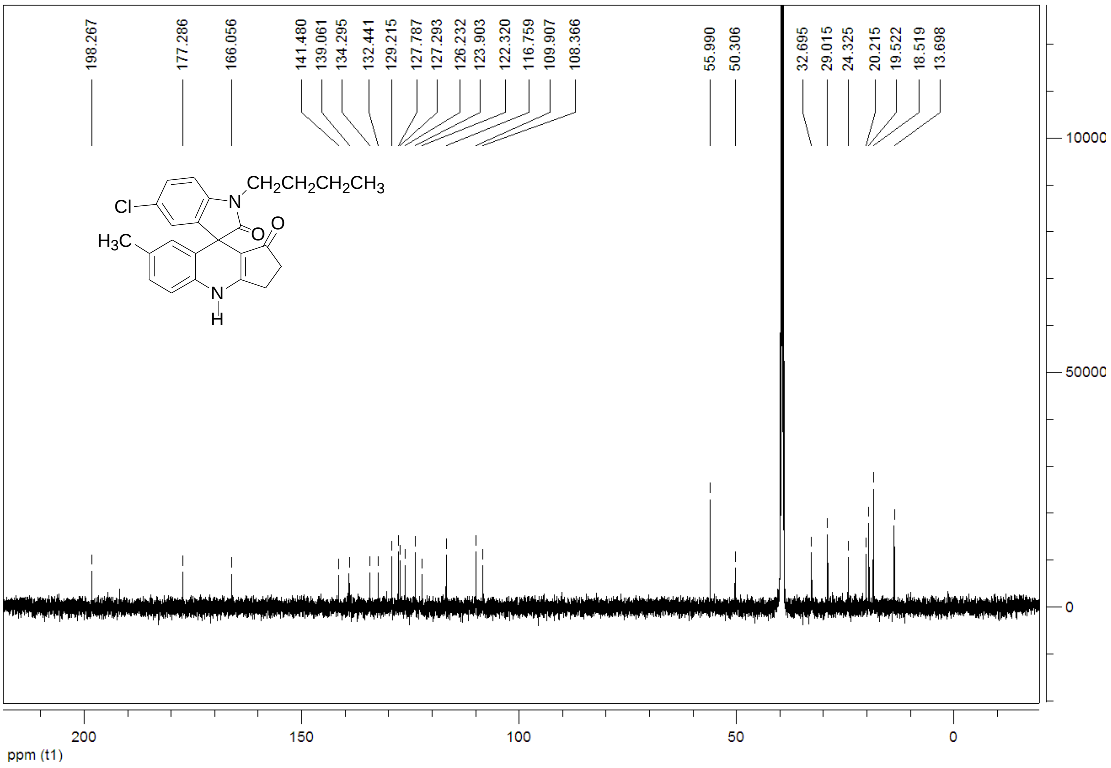

1j: white solid, 58%, mp 286–288 °C; ^{1}H NMR (600 MHz, $CDCl_3$) δ: 10.32 (s, 1H, NH), 7.10–7.08 (m, 1H, ArH), 7.07–7.04 (m, 1H, ArH), 7.01–7.00 (m, 1H, ArH), 6.93 (d, *J* = 8.4 Hz, 1H, ArH), 6.74–6.73 (m, 1H, ArH), 6.20 (s, 1H, ArH), 3.72–3.71 (m, 2H, CH_2), 2.75–2.74 (m, 2H, CH_2), 2.22–2.21 (m, 2H, CH_2), 2.04 (s, 3H, CH_3), 1.65–1.62 (m, 2H, CH_2), 1.40–1.36 (m, 2H, CH_2), 0.91 (t, *J* = 7.8 Hz, 3H, CH_3); ^{13}C NMR (150 MHz, $CDCl_3$) δ: 198.2, 177.5, 166.0, 159.4, 157.8, 138.8, 134.3, 132.4, 129.1, 127.3, 122.5, 116.7, 114.1, 114.0, 113.9, 111.7, 111.6, 109.2, 108.5, 56.0, 50.6, 32.7, 29.0, 24.3, 20.2, 19.5, 18.5, 13.7; IR (KBr) υ: 3231, 3171, 3102, 3049, 2961, 2868, 1684, 1605, 1533, 1493, 1449, 1377, 1345, 1255, 1184, 1125, 1045, 996, 869, 818 cm^{-1}; MS (*m*/*z*): HRMS (ESI) Calcd. for $C_{24}H_{22}FN_2O_2$ ($[M-H]^-$): 387.1671. Found: 389.1666.

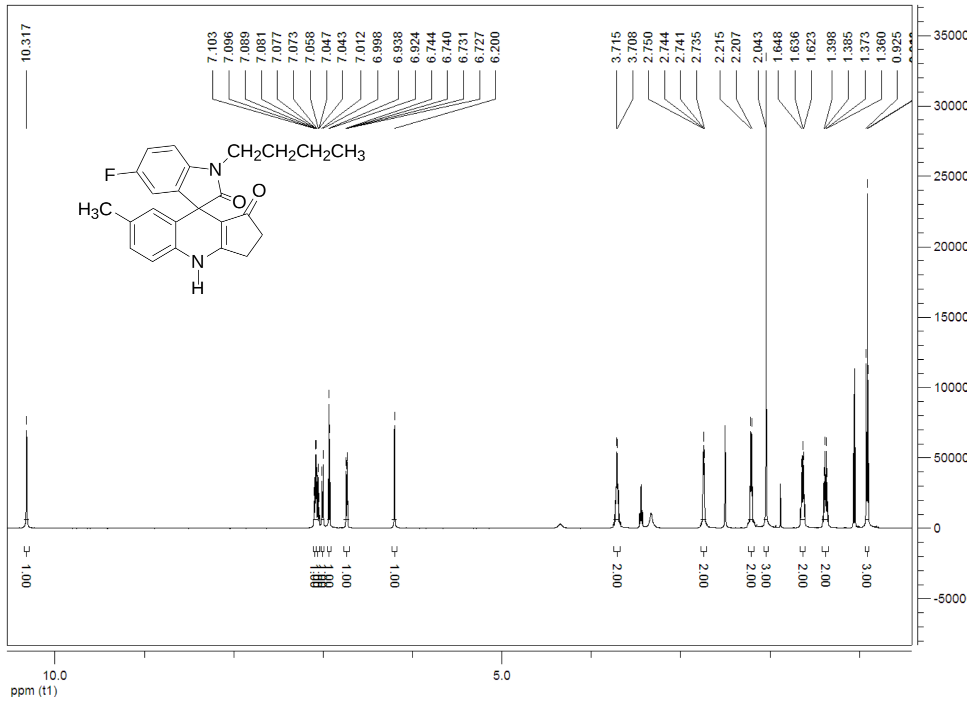

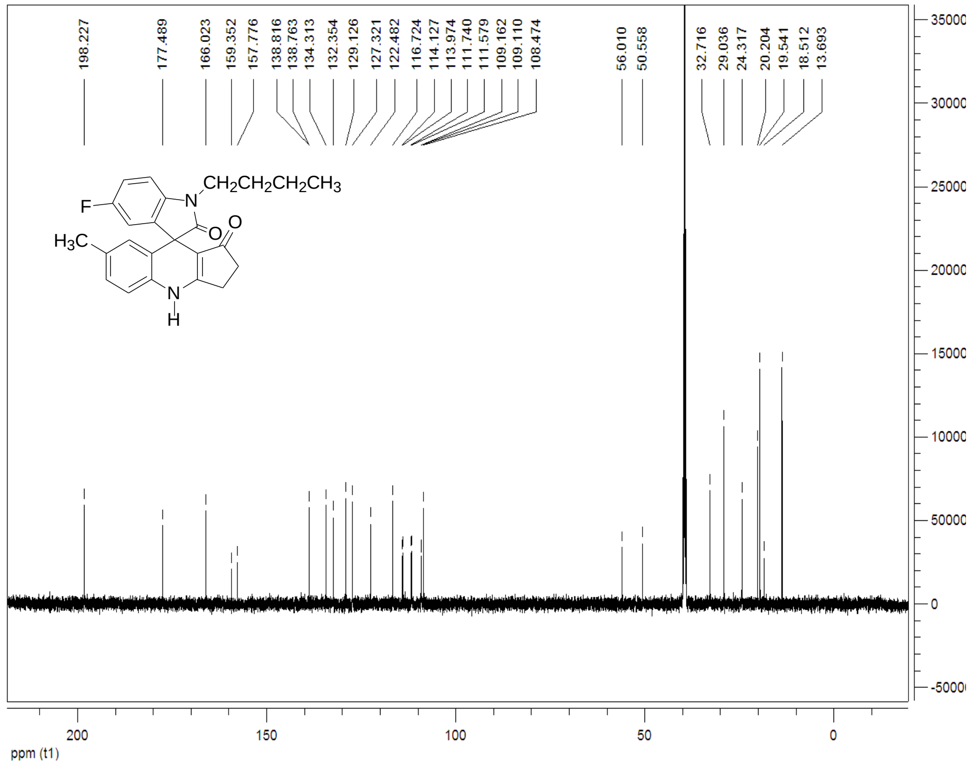

1k: white solid, 79%, mp >300 °C; ^{1}H NMR (600 MHz, $CDCl_3$) δ: 10.31 (s, 1H, NH), 9.06 (s, 1H, OH), 7.02 (d, *J* = 7.8 Hz, 1H, ArH), 6.95 (d, *J* = 7.8 Hz, 1H, ArH), 6.85 (d, *J* = 8.4 Hz, 1H, ArH), 6.66 (s, 1H, ArH), 6.61–6.59 (m, 2H, ArH), 5.84 (d, *J* = 2.4 Hz, 1H, ArH), 3.68–3.66 (m, 2H, CH_2), 2.74–2.70 (m, 2H, CH_2), 2.21–2.18 (m, 2H, CH_2), 2.16 (s, 3H, CH_3), 1.65–1.61 (m, 2H, CH_2), 1.41–1.38 (m, 2H, CH_2), 0.92 (t, *J* = 7.2 Hz, 3H, CH_3); ^{13}C NMR (150 MHz, $CDCl_3$) δ: 198.0, 177.5, 165.5, 153.4, 140.1, 137.5, 131.2, 128.9, 128.0, 124.4, 124.3, 118.7, 117.5, 115.7, 115.6, 113.4, 108.0, 107.9, 50.5, 32.7, 29.2, 24.2, 21.0, 20.5, 19.6, 13.8; IR (KBr) υ: 3040, 2967, 2927, 1673, 1591, 1543, 1491, 1384, 1340, 1298, 1266, 1214, 1163, 1122, 1043, 864, 819 cm^{-1}; MS (*m*/*z*): HRMS (ESI) Calcd. for $C_{24}H_{23}N_2O_3$ ($[M-H]^-$): 387.1714. Found: 387.1708.

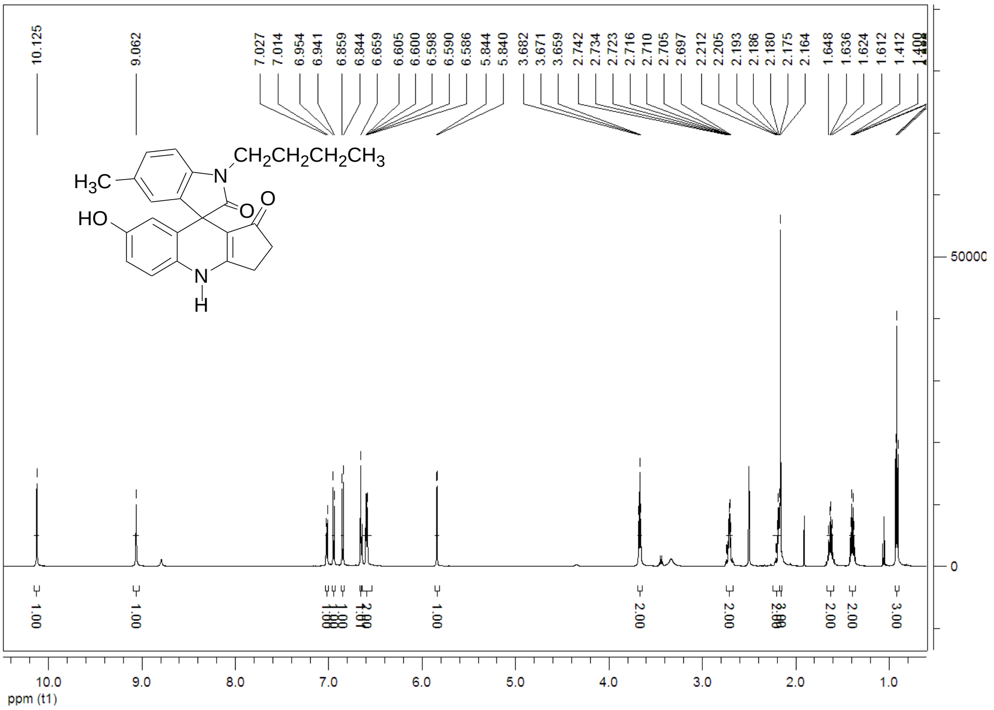

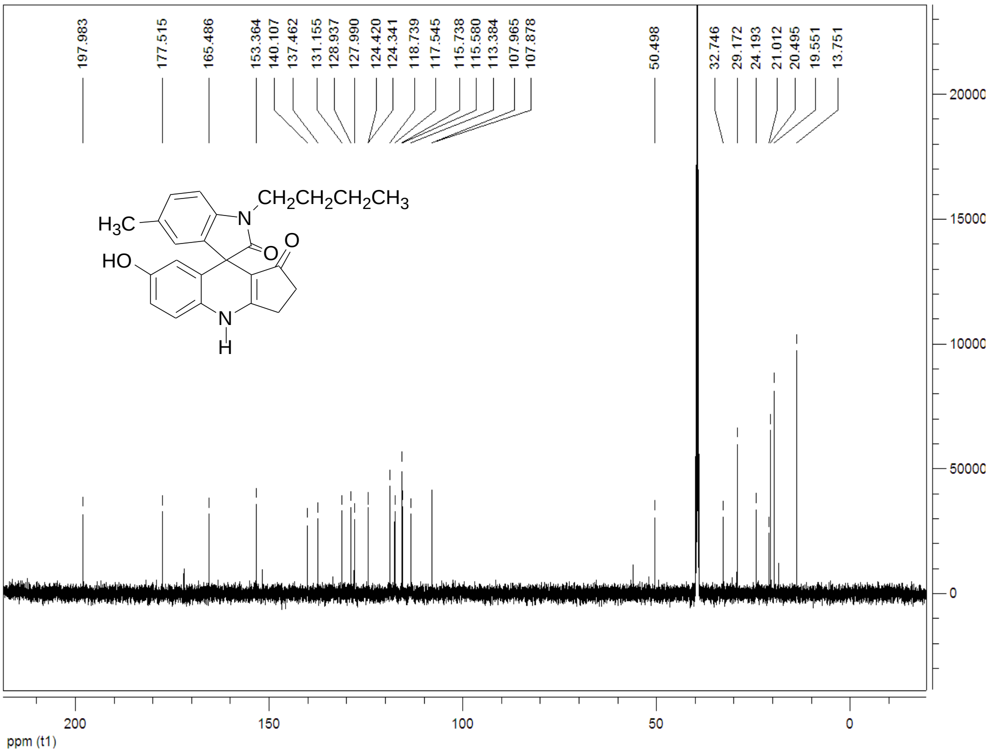

1l: white solid, 67%, mp >300 °C; ^{1}H NMR (600 MHz, DMSO-d_6) δ: 10.44 (s, 1H, NH), 8.47 (d, J = 8.4 Hz, 1H, ArH), 7.83 (d, J = 7.8 Hz, 1H, ArH), 7.66 (t, J = 7.8 Hz, 1H, ArH), 7.58–7.55 (m, 3H, ArH), 7.40–7.35 (m, 3H, ArH), 7.29 (t, J = 7.2 Hz, 1H, ArH), 6.95 (d, J = 8.4 Hz, 1H, ArH),6.74 (d, J = 7.8 Hz, 2H, ArH), 6.50 (d, J = 8.4 Hz, 1H, ArH), 5.07 (d, J = 16.2 Hz, 1H, CH), 4.92 (d, J = 16.2 Hz, 1H, CH), 2.96–2.93 (m, 2H, CH_2), 2.36–2.22 (m, 2H, CH_2), 2.10 (s, 3H, CH_3); ^{13}C NMR (150 MHz, DMSO-d_6) δ: 199.2, 177.9, 166.4, 139.5, 137.5, 136.4, 132.8, 131.8, 131.7, 128.4, 128.3, 128.1, 127.3, 127.2, 126.6, 126.4, 124.9, 124.8, 123.3, 122.7, 121.3, 118.3, 110.4, 108.7, 56.0, 50.9, 43.2, 33.0, 25.0, 20.4, 18.5; IR (KBr) υ: 3245, 3055, 2964, 2922, 1689, 1617, 1525, 1438, 1362, 1248, 1192, 1162, 1056, 1034, 888, 809 cm^{-1}; MS (m/z): HRMS (ESI) Calcd. for $C_{31}H_{23}N_2O_2$ ($[M-H]^-$): 455.1765. Found: 455.1758.

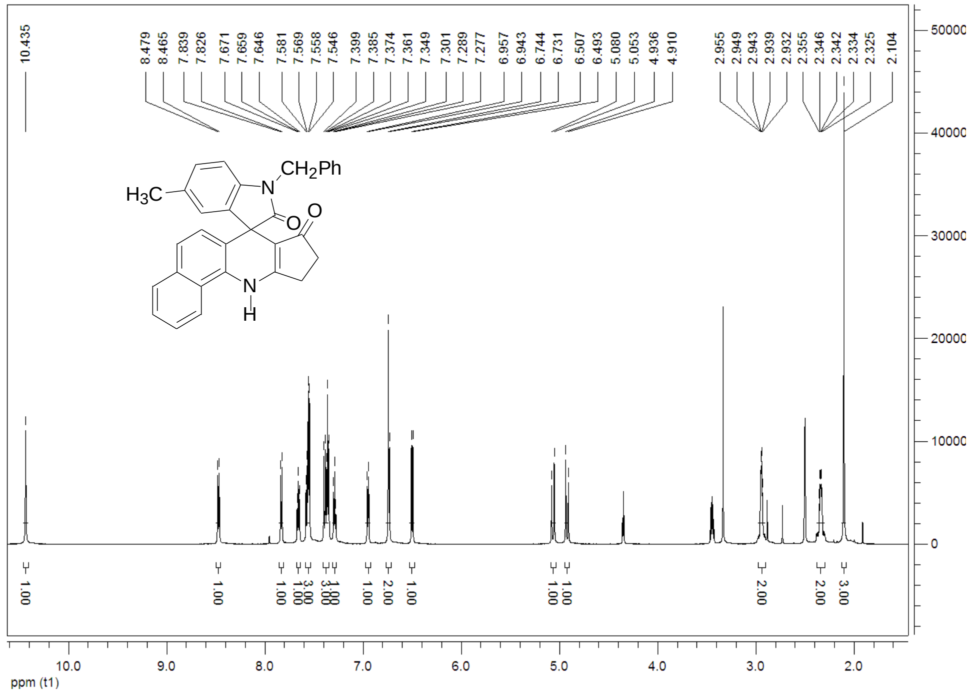

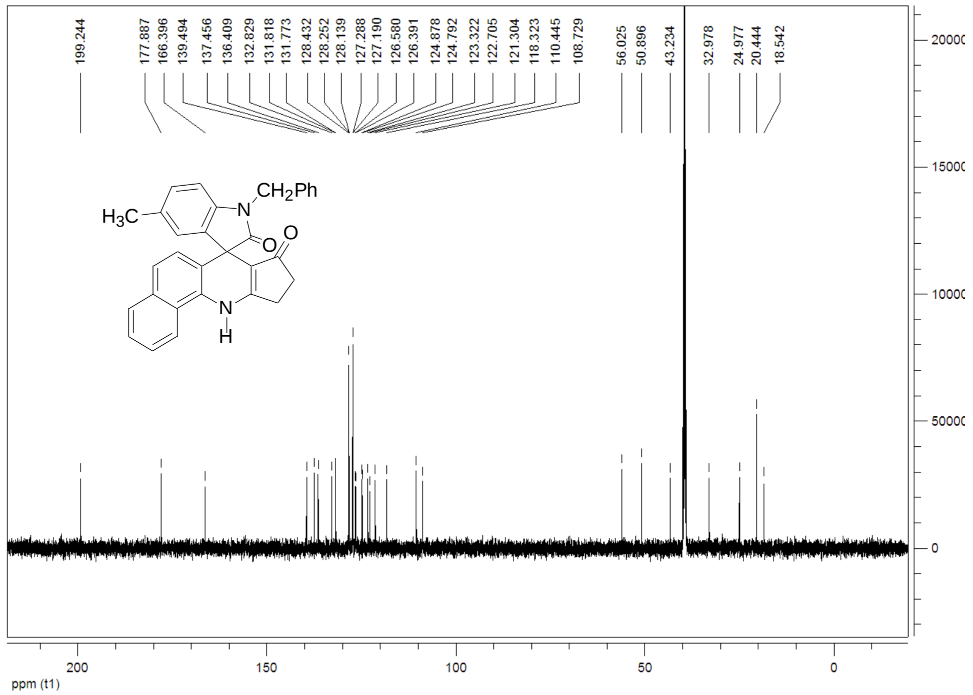

2a: white solid, 35%, mp >300 °C; ^{1}H NMR (600 MHz, DMSO-d_6) δ: 10.41 (s, 1H, NH), 10.21 (s, 1H, NH), 7.15–7.13 (m, 1H, ArH), 6.97 (d, J = 8.4 Hz, 1H, ArH), 6.87 (d, J = 7.8 Hz, 1H, ArH), 6.84–6.82 (m, 2H, ArH), 6.80 (d, J = 6.6 Hz, 1H, ArH), 6.61 (s, 1H, ArH), 5.99 (d, J = 3.0 Hz, 1H, ArH), 3.54 (s, 3H, OCH_3), 2.74–2.72 (m, 2H, CH_2), 2.22–2.20 (m, 2H, CH_2); ^{13}C NMR (150 MHz, DMSO-d_6) δ: 198.2, 179.2, 165.7, 155.3, 141.4, 137.9, 130.3, 127.7, 124.6, 123.9, 121.8, 117.4, 113.3, 112.9, 109.2, 108.1, 55.2, 50.9, 32.8, 24.2; IR (KBr) υ: 3203, 3108, 2966, 2925, 2833, 1703, 1661, 1590, 1538, 1494, 1388, 1330, 1288, 1251, 1194, 1162, 1124, 1039, 911, 857, 805 cm^{-1}; MS (m/z): HRMS (ESI) Calcd. for $C_{20}H_{15}N_2O_3$ ($[M-H]^-$): 331.1088. Found: 331.1088.

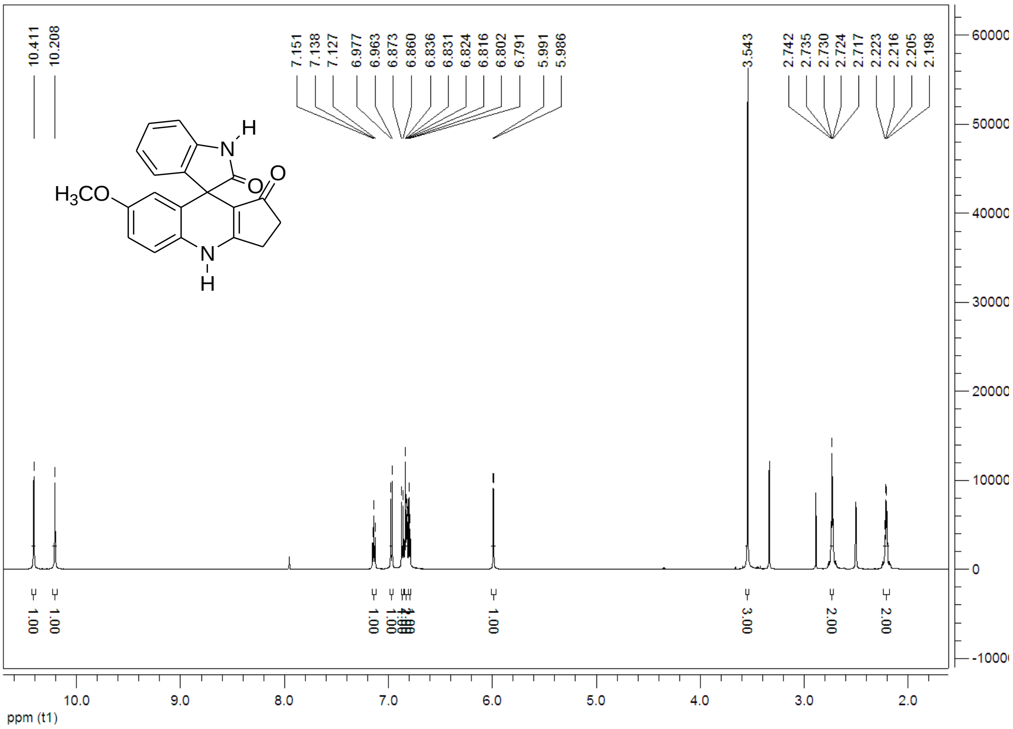

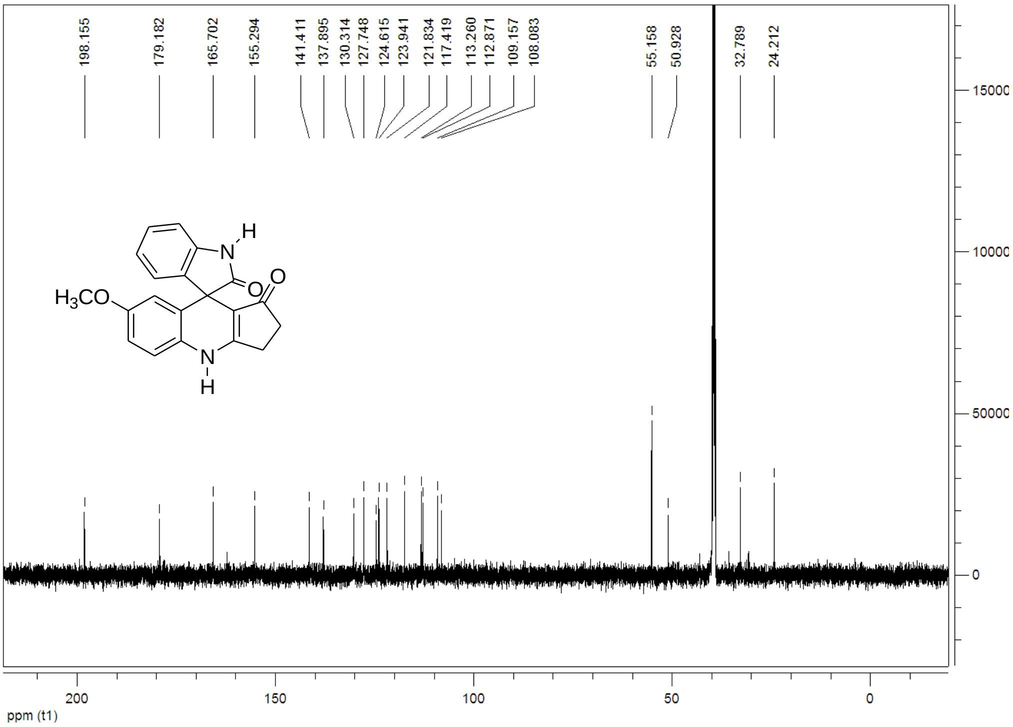

2b: white solid, 36%, mp >300 °C; ^{1}H NMR (600 MHz, DMSO-d_6) δ: 10.31 (s, 1H, NH), 10.19 (s, 1H, NH), 6.96 (d, J = 8.4 Hz, 1H, ArH), 6.94 (d, J = 7.8 Hz, 1H, ArH), 6.83–6.81 (m, 1H, ArH), 6.75 (d, J = 7.8 Hz, 1H, ArH), 6.61 (s, 1H, ArH), 5.99 (d, J = 2.4 Hz, 1H, ArH), 3.55 (s, 3H, OCH_3), 2.73–2.71 (m, 2H, CH_2), 2.22–2.21 (m, 2H, CH_2), 2.13 (s, 3H, CH_3); ^{13}C NMR (150 MHz, DMSO-d_6) δ: 198.2, 179.2, 165.7, 155.3, 138.9, 138.1, 130.6, 130.3, 128.0, 124.8, 124.5, 117.4, 113.2, 113.0, 108.9, 108.2, 55.1, 51.0, 32.8, 24.2, 20.5; IR (KBr) υ: 3229, 3109, 3054, 2959, 2914, 2834, 1698, 1669, 1600, 1539, 1494, 1381, 1326, 1288, 1240, 1158, 1116, 1033, 959, 861, 807 cm^{-1}; MS (m/z): HRMS (ESI) Calcd. for $C_{21}H_{17}N_2O_4$ ($[M-H]^-$): 345.1245. Found: 345.1242.

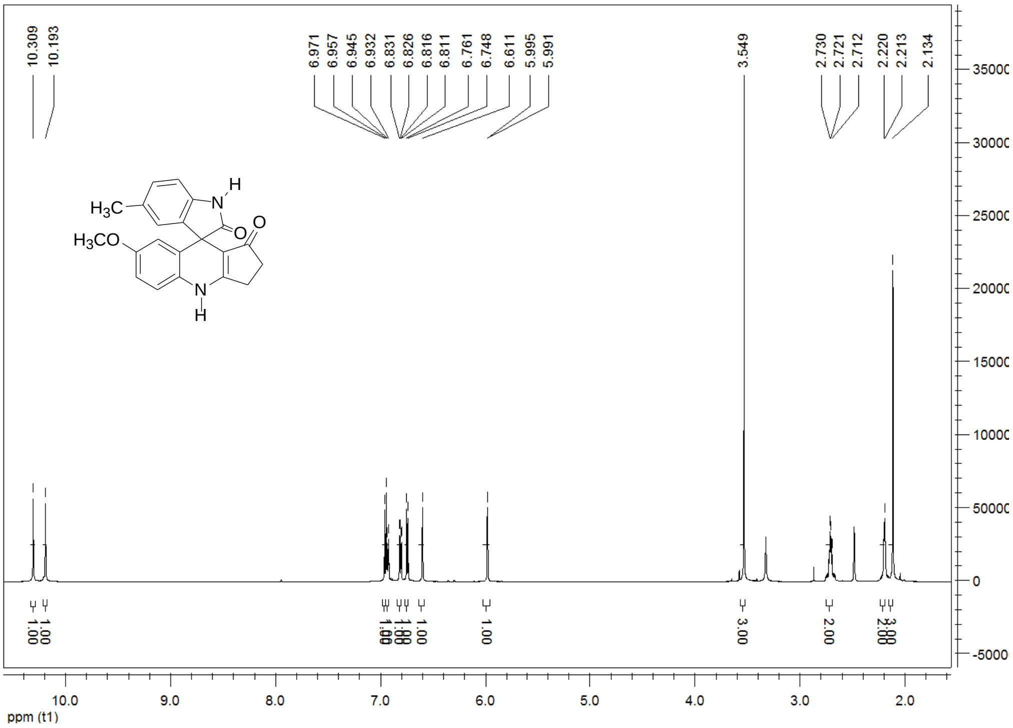

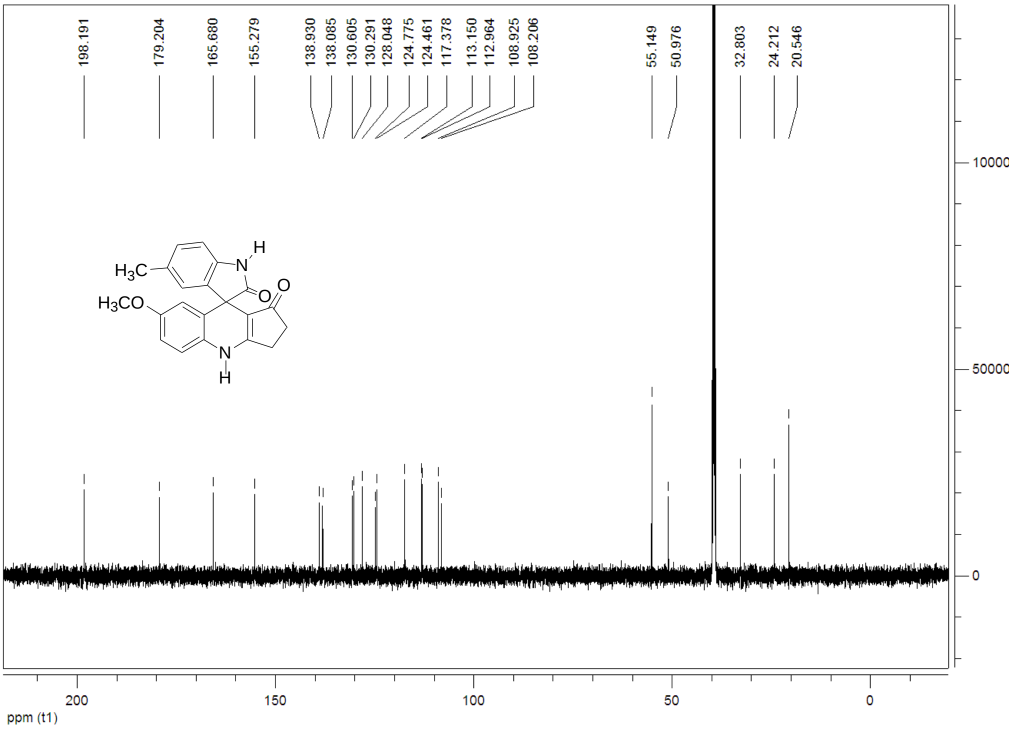

2c: white solid, 35%, mp >300 °C; ^{1}H NMR (600 MHz, DMSO-d_6) δ: 10.57 (s, 1H, NH), 10.28 (s, 1H, NH), 7.21–7.19 (m, 1H, ArH), 7.00 (d, J = 9.0 Hz, 1H, ArH), 6.89 (d, J = 8.4 Hz, 1H, ArH), 6.87–6.85 (m, 1H, ArH), 6.83–6.82 (m, 1H, ArH), 5.99 (d, J = 2.4 Hz, 1H, ArH), 3.56 (s, 3H, OCH_3), 2.75–2.73 (m, 2H, CH_2), 2.24–2.22 (m, 2H, CH_2); ^{13}C NMR (150 MHz, DMSO-d_6) δ: 198.3, 178.9, 166.0, 155.4, 140.5, 139.5, 130.3, 127.8, 125.7, 123.9, 123.8, 117.7, 113.6, 112.7, 110.7, 107.5, 55.2, 51.1, 32.8, 24.3; IR (KBr) υ: 3210, 3058, 2967, 2918, 2834, 1703, 1670, 1600, 1539, 1493, 1438, 1329, 1286, 1241, 1188, 1122, 1035, 956, 871, 806 cm^{-1}; MS (m/z): HRMS (ESI) Calcd. for $C_{20}H_{14}N_2O_3$ ([M-H]$^-$): 365.0698. Found: 365.0697.

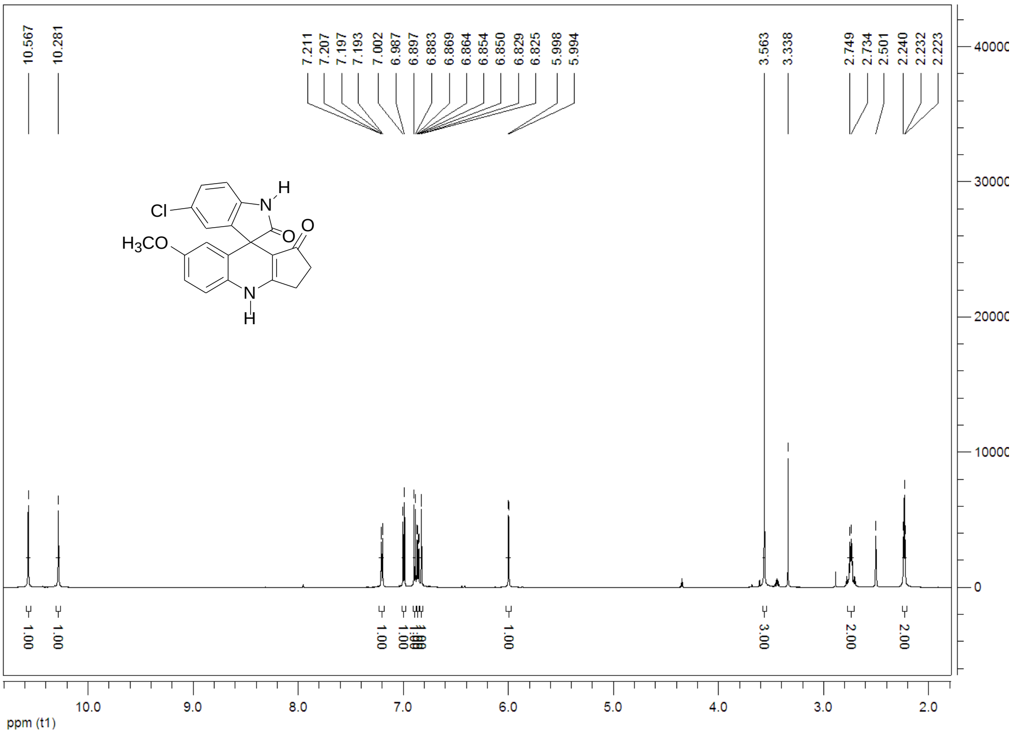

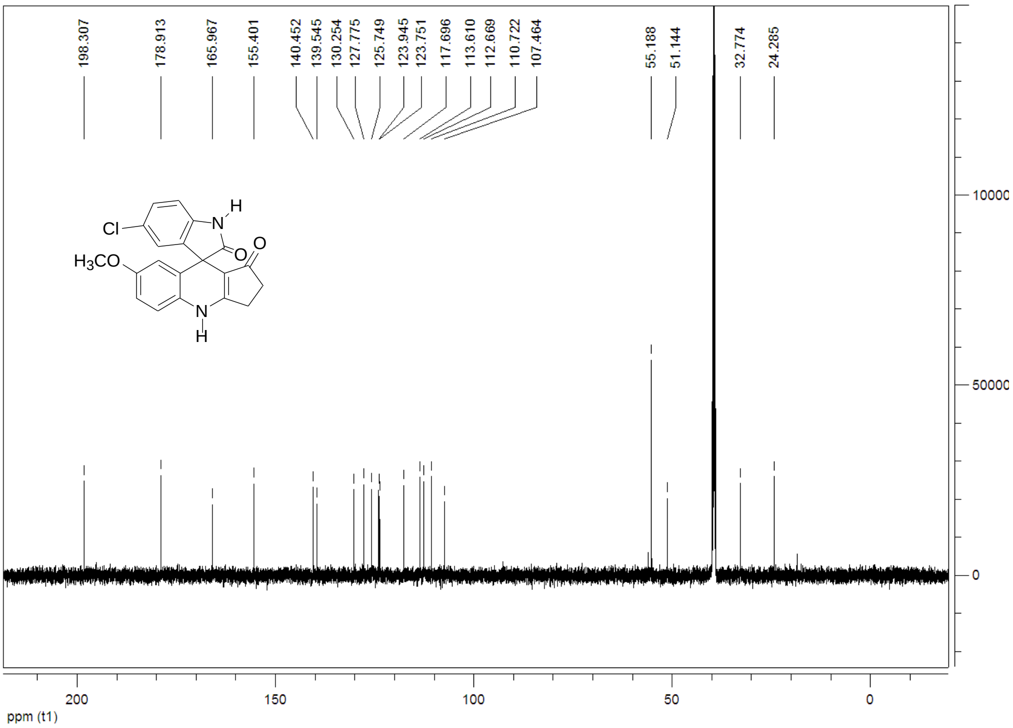

2d: white solid, 30%, mp >300 °C; ^{1}H NMR (600 MHz, DMSO-d_6) δ: 10.44 (s, 1H, NH), 10.26 (s, 1H, NH), 6.99–6.96 (m, 2H, ArH), 6.87–6.84 (m, 2H, ArH), 6.69–6.67 (m, 1H, ArH), 5.99 (d, J = 2.4 Hz, 1H, ArH), 3.56 (s, 3H, OCH_3), 2.74–2.73 (m, 2H, CH_2), 2.23–2.22 (m, 2H, CH_2); ^{13}C NMR (150 MHz, DMSO-d_6) δ: 198.2, 179.2, 165.9, 159.0, 157.4, 155.4, 139.2, 139.1, 137.7, 130.3, 123.9, 117.6, 114.2, 114.0, 113.6, 112.7, 111.7, 111.5, 110.0, 109.9, 107.6, 55.2, 55.1, 32.8, 24.3; IR (KBr) υ: 3202, 3109, 3069, 2971, 2922, 1700, 1659, 1589, 1542, 1490, ,1385, 1332, 1291, 1253, 1195, 1159, 1116, 1039, 882, 803 cm^{-1}; MS (m/z): HRMS (ESI) Calcd. for $C_{20}H_{14}FN_2O_3$ ([M-H]$^-$): 349.0994. Found: 349.0996.

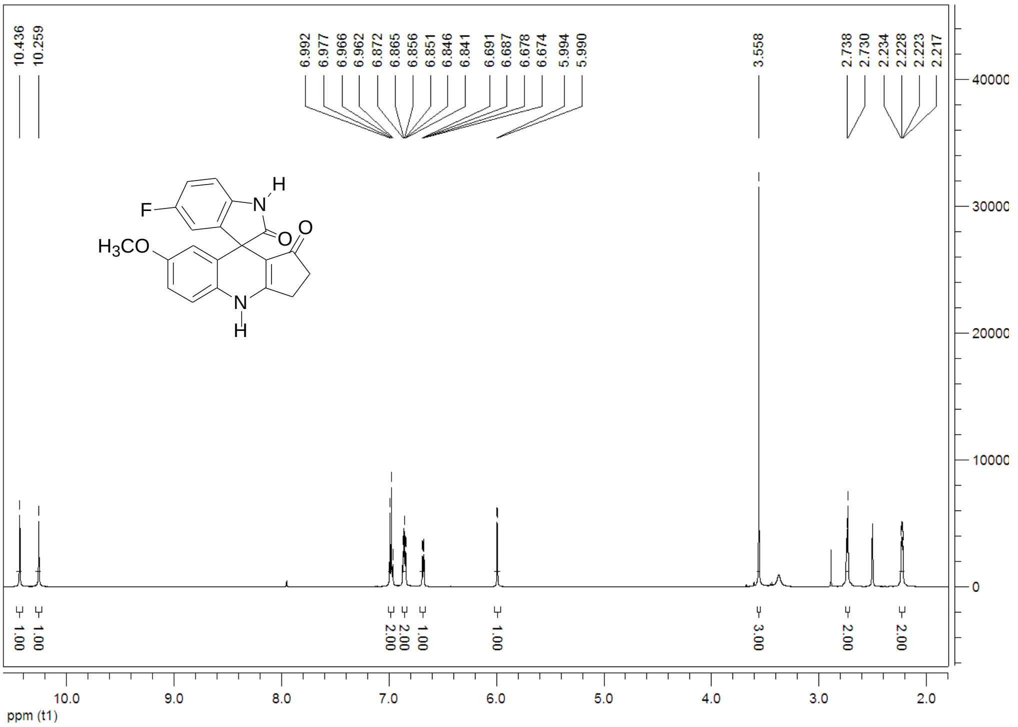

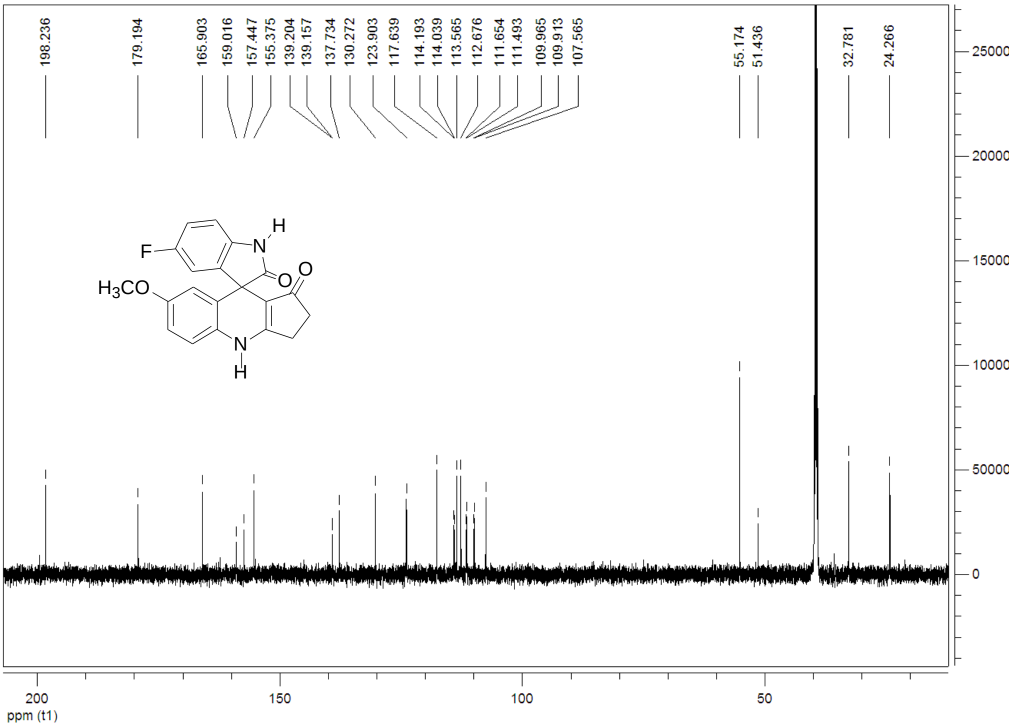

2e: white solid, 26%, mp >300 °C; ^{1}H NMR (600 MHz, DMSO-d_6) δ: 10.39 (s, 1H, NH), 10.22 (s, 1H, NH), 7.13 (t, *J* = 7.2 Hz, 1H, ArH), 6.98 (d, *J* = 8.4 Hz, 1H, ArH), 6.91 (d, *J* = 7.8 Hz, 1H, ArH), 6.86 (d, *J* = 7.2 Hz, 1H, ArH), 6.83 (t, *J* = 7.8 Hz, 1H, ArH), 6.78 (d, *J* = 7.2 Hz, 1H, ArH), 6.30 (s, 1H, ArH), 2.73 (brs, 2H, CH_2), 2.21–2.20 (m, 2H, CH_2), 2.06 (s, 3H, CH_3); ^{13}C NMR (150 MHz, DMSO-d_6) δ: 198.3, 179.3, 165.8, 141.4, 138.2, 134.3, 132.2, 128.8, 127.7, 127.5, 124.0, 123.3, 121.8, 116.5, 109.2, 50.7, 32.8, 24.2, 20.3; IR (KBr) υ: 3241, 3172, 1700, 1663, 1599, 1531, 1496, 1469, 1393, 1328, 1231, 1190, 1113, 1049, 822, 758 cm^{-1}; MS (*m*/*z*): HRMS (ESI) Calcd. for $C_{20}H_{15}N_2O_2$ ($[M-H]^-$): 315.1139. Found: 315.1138.

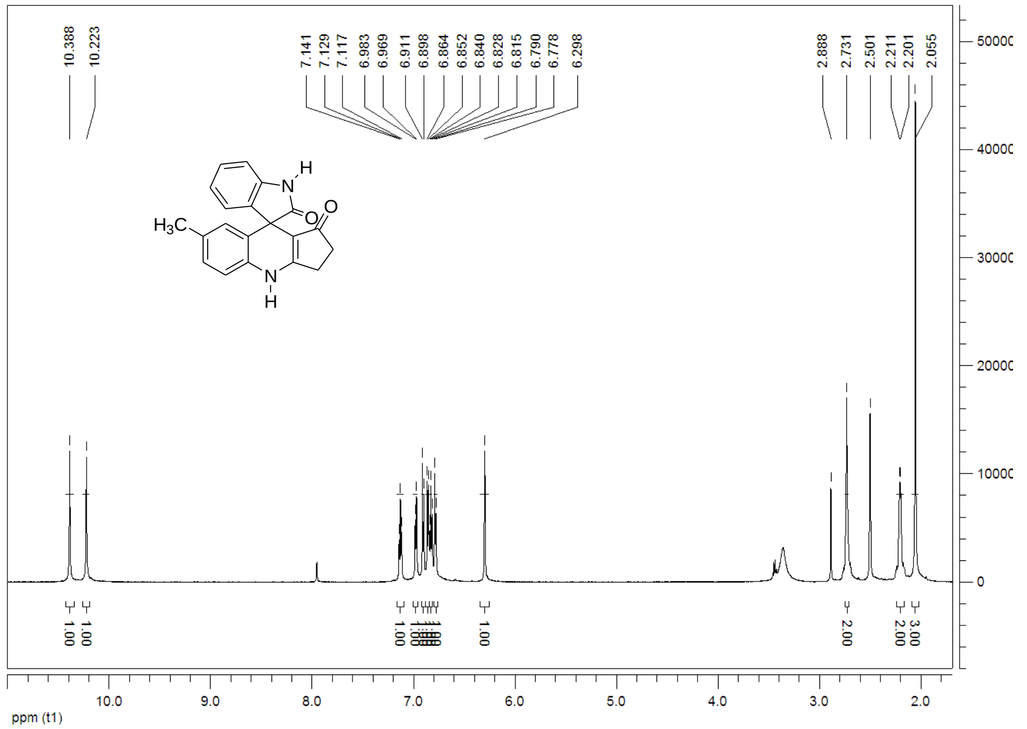

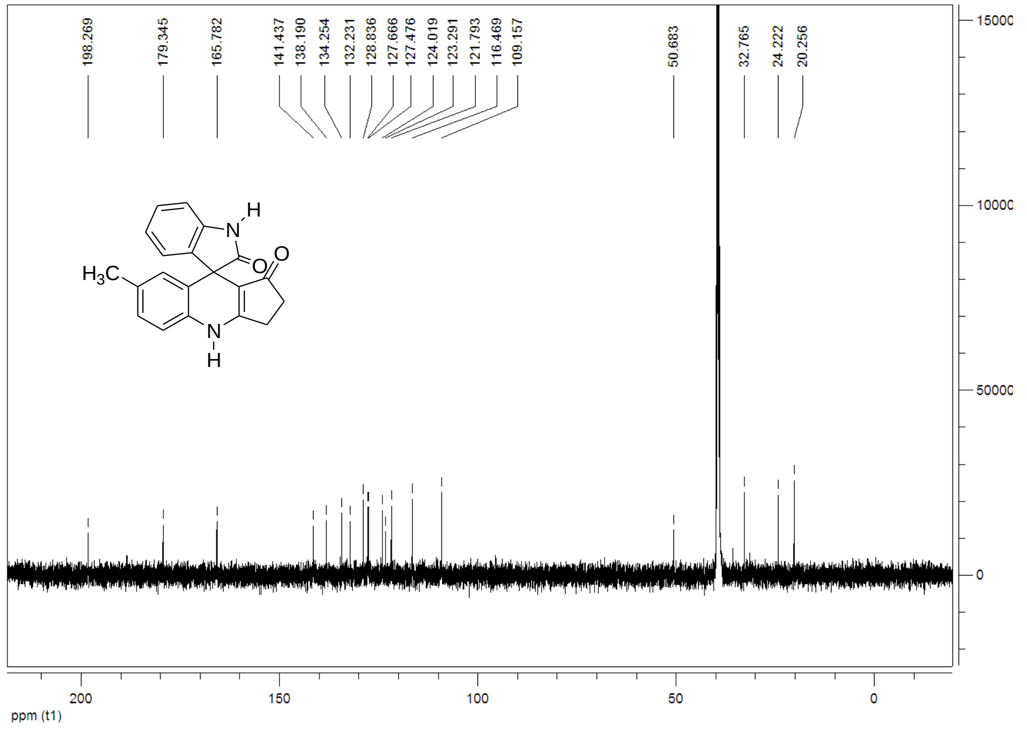

2f: white solid, 35%, mp >300 °C; ^{1}H NMR (600 MHz, DMSO-d_6) δ: 10.29 (s, 1H, NH), 10.21 (s, 1H, NH), 6.98–6.96 (m, 1H, ArH), 6.93 (d, J = 7.8 Hz, 1H, ArH), 6.90 (d, J = 7.8 Hz, 1H, ArH), 6.75 (d, J = 7.8 Hz, 1H, ArH), 6.60 (s, 1H, ArH), 6.30 (s, 1H, ArH), 2.73–2.72 (m, 2H, CH_2), 2.22–2.20 (m, 2H, CH_2), 2.13 (s, 3H, CH_3), 2.06 (s, 3H, CH_3); ^{13}C NMR (150 MHz, DMSO-d_6) δ: 198.3, 179.4, 165.8, 139.0, 138.4, 134.2, 132.2, 130.5, 128.8, 128.0, 127.5, 124.5, 123.4, 116.4, 109.3, 108.9, 50.7, 35.7, 32.8, 30.7, 24.2, 20.6, 20.3; IR (KBr) υ: 3231, 2919, 1698, 1602, 1532, 1492, 1380, 1298, 1240, 1094, 1049, 816 cm^{-1}; MS (m/z): HRMS (ESI) Calcd. for $C_{21}H_{17}N_2O_2$ ($[M-H]^-$): 329.1296. Found: 329.1294.

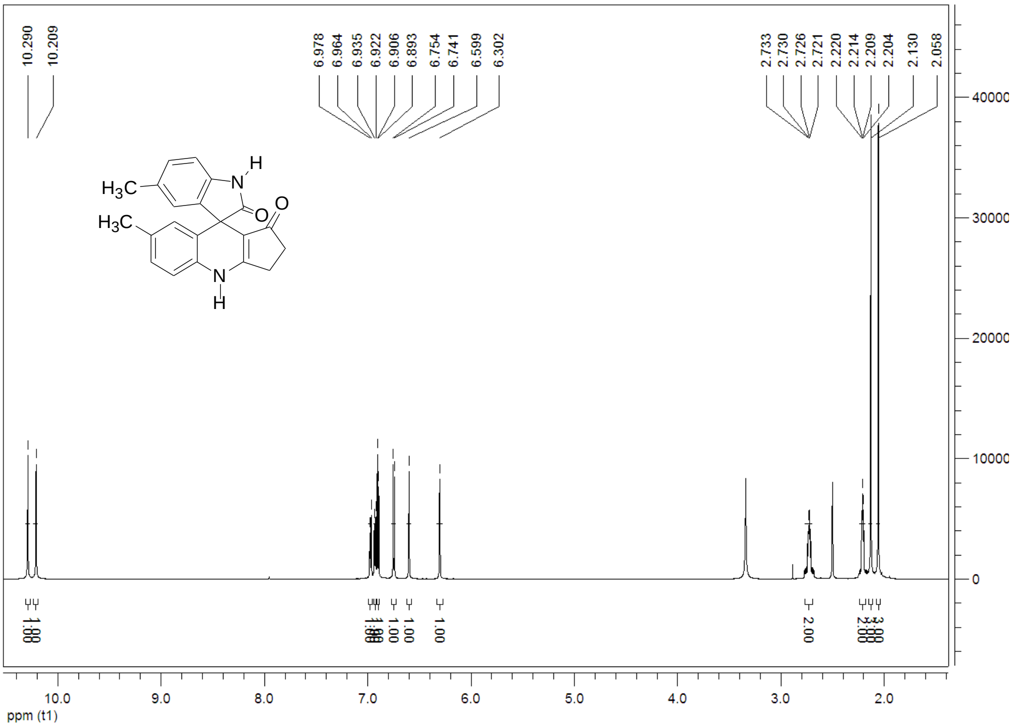

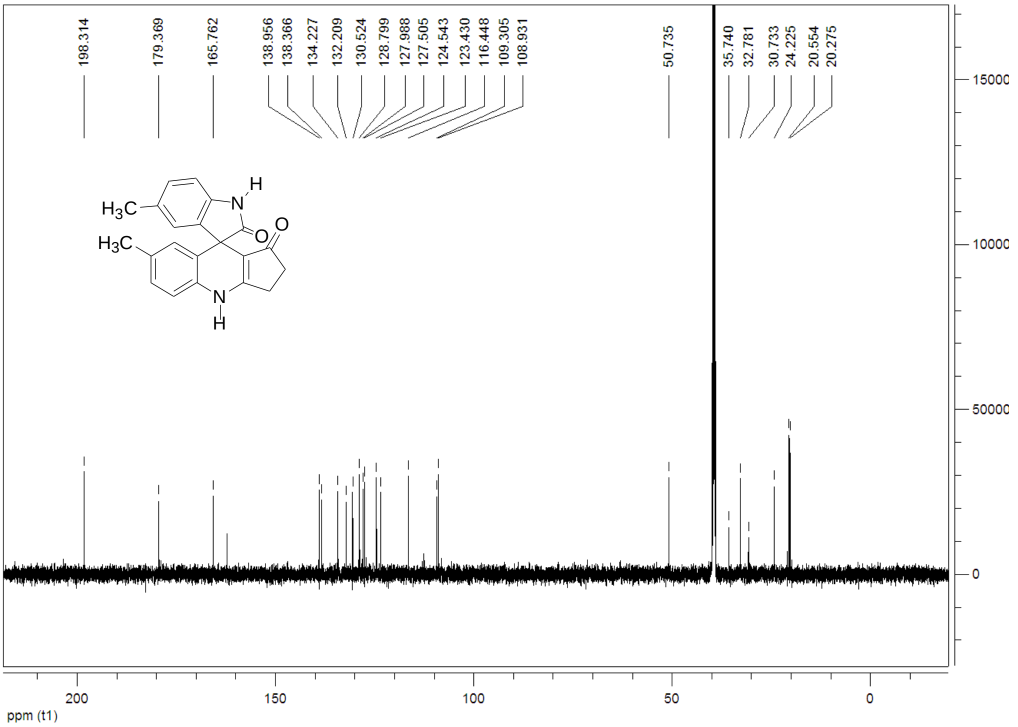

2g: white solid, 32%, mp >300 °C; ^{1}H NMR (600 MHz, DMSO-d_6) δ: 10.55 (s, 1H, NH), 10.30 (s, 1H, NH), 7.20–7.18 (m, 1H, ArH), 7.01 (d, J = 7.8 Hz, 1H, ArH), 6.93 (d, J = 8.4 Hz, 1H, ArH), 6.89 (d, J = 8.4 Hz, 1H, ArH), 6.81 (d, J = 1.8 Hz, 1H, ArH), 6.32 (s, 1H, ArH), 2.76–2.74 (m, 2H, CH_2), 2.24–2.22 (m, 2H, CH_2), 2.07 (s, 3H, CH_3); ^{13}C NMR (150 MHz, DMSO-d_6) δ: 198.4, 179.1, 166.1, 162.3, 140.5, 139.8, 134.2, 132.5, 129.2, 127.7, 127.4, 125.7, 124.0, 122.5, 116.7, 110.7, 108.5, 50.9, 35.7, 32.8, 30.7, 24.3, 20.2; IR (KBr) υ: 3384, 3240, 3168, 3095, 2923, 2846, 1701, 1599, 1531, 1491, 1380, 1329, 1295, 1239, 1188, 1094, 1050, 956, 877, 819 cm^{-1}; MS (m/z): HRMS (ESI) Calcd. for $C_{20}H_{14}ClN_2O_2$ ($[M-H]^-$): 349.0749. Found: 349.0747.

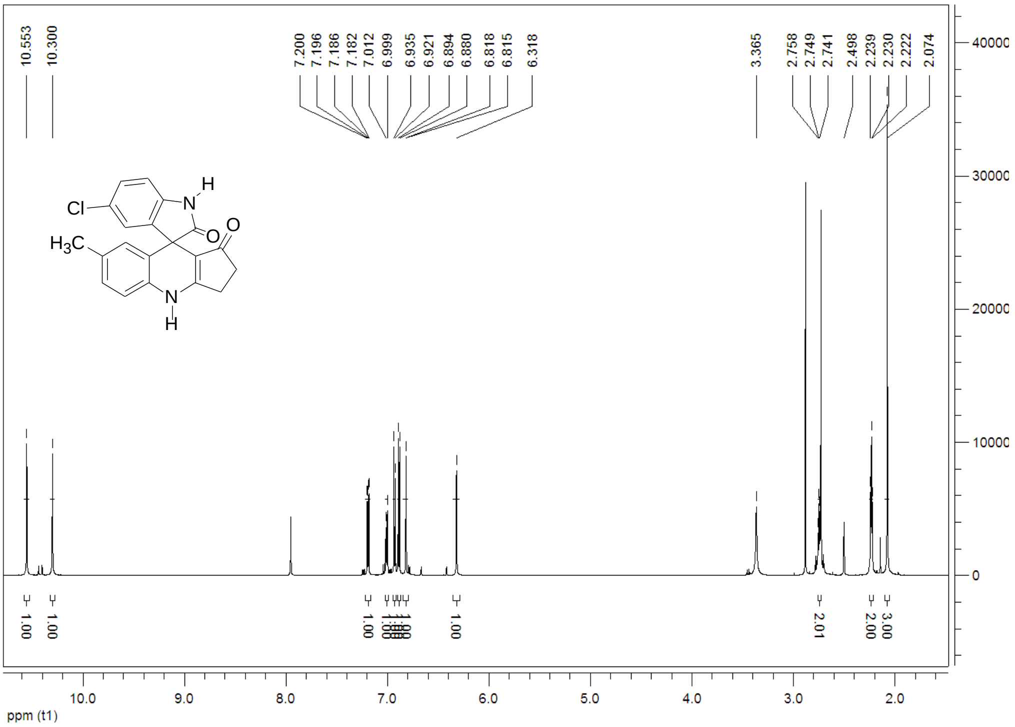

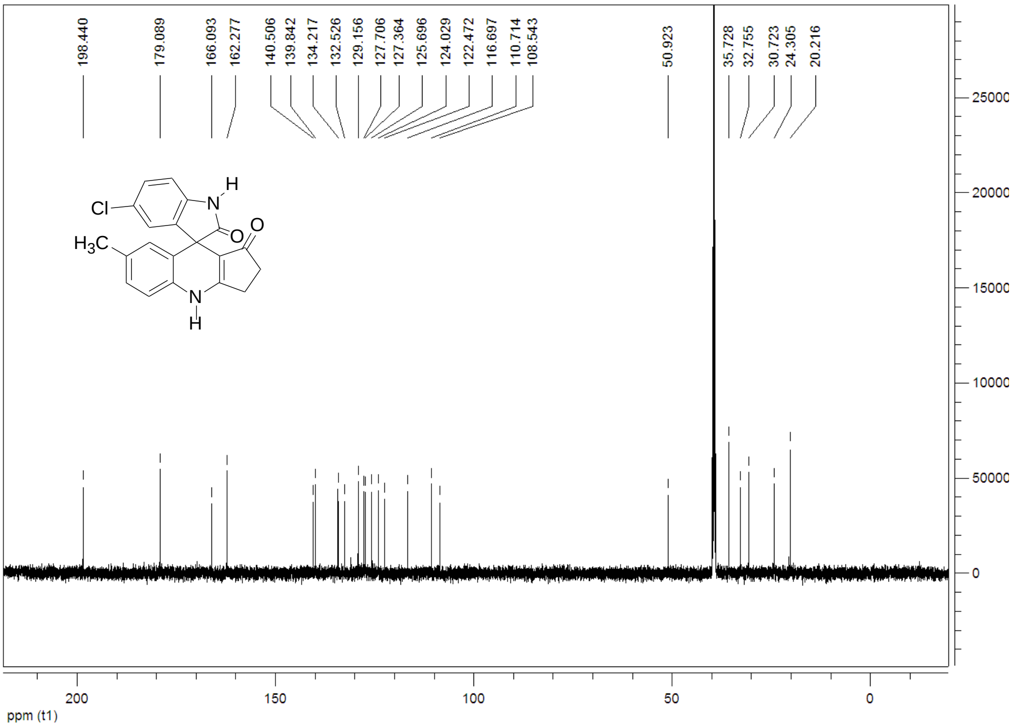

3a: white solid, 76%, mp 239–241 °C; ^{1}H NMR (600 MHz, DMSO-d_6) δ: 11.57 (brs, 2H, OH), 10.42 (s, 1H, NH), 7.23 (d, *J* = 6.6 Hz, 1H, ArH), 7.02 (brs, 1H, ArH), 6.75 (brs, 1H, ArH), 6.68 (d, *J* = 7.2 Hz, 1H, ArH), 2.28 (brs, 8H, CH_2); ^{13}C NMR (150 MHz, DMSO-d_6) δ: 179.0, 141.6, 132.7, 132.6, 126.8, 126.7, 125.7, 120.6, 112.4, 108.5, 43.3, 30.4, 30.3, 30.2, 30.1, 30.0, 29.9, 29.8, 29.7; IR (KBr) υ: 3196, 2925, 1676, 1612, 1573, 1478, 1435, 1385, 1362, 1306, 1232, 1110, 1069, 1040, 881 cm^{-1}; MS (*m/z*): HRMS (ESI) Calcd. for $C_{18}H_{14}NO_5$ ($[M-H]^-$): 324.0877 Found: 324.0874.

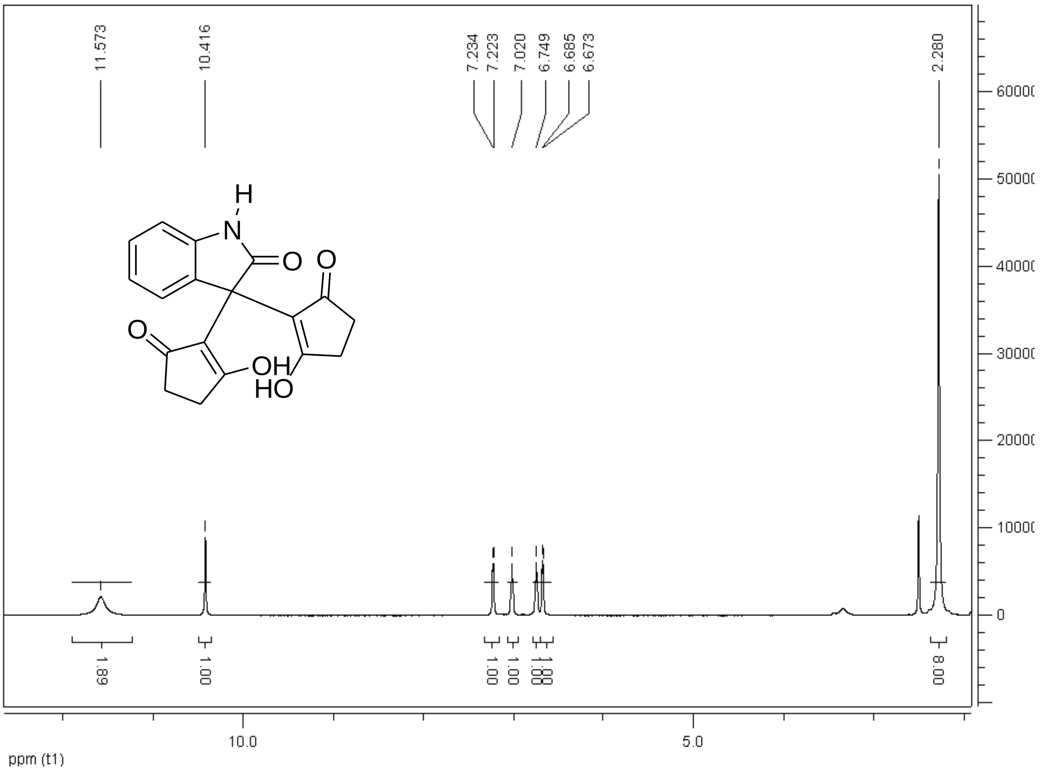

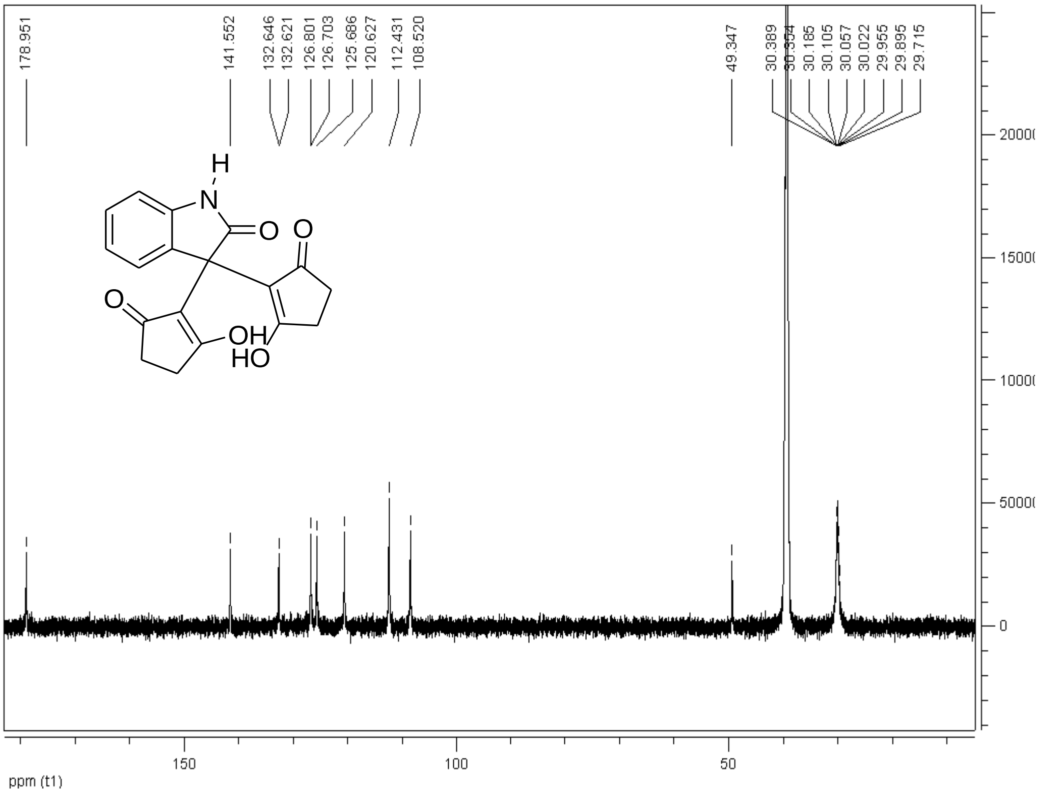

3b: white solid, 80%, mp 252–254 °C; ^{1}H NMR (600 MHz, DMSO-d_6) δ: 11.51 (brs, 2H, OH), 10.34 (s, 1H, NH), 7.06 (s, 1H, ArH), 6.83 (d, *J* = 7.8 Hz, 1H, ArH), 6.57 (d, *J* = 7.8 Hz, 1H, ArH), 2.29 (brs, 8H, CH_2), 2.15 (s, 3H, CH_3); ^{13}C NMR (150 MHz, DMSO-d_6) δ: 178.9, 139.2, 123.6, 129.0, 127.8, 126.4, 112.5, 108.2, 40.4, 20.9; IR (KBr) υ: 3198, 2922, 1673, 1611, 1574, 1494, 1435, 1364, 1308, 1139, 1071, 890, 851, 813 cm^{-1}; MS (*m/z*): HRMS (ESI) Calcd. for $C_{19}H_{16}NO_5$ ([M-H]$^-$): 338.1034 Found: 338.1033.

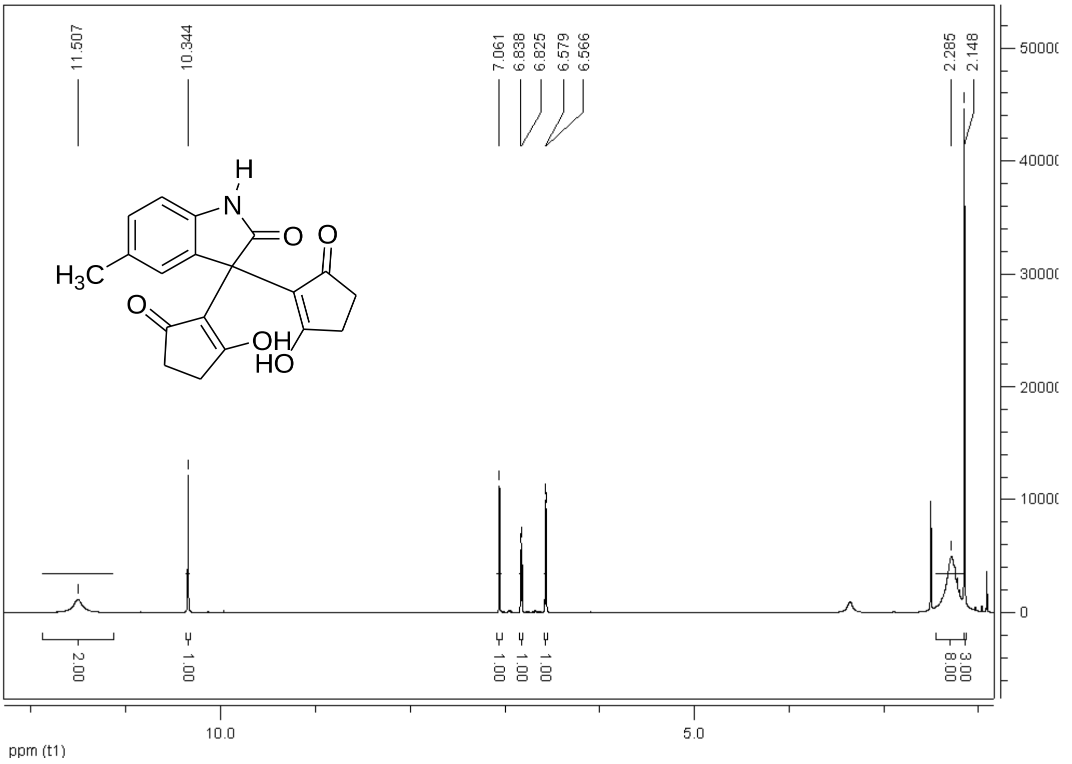

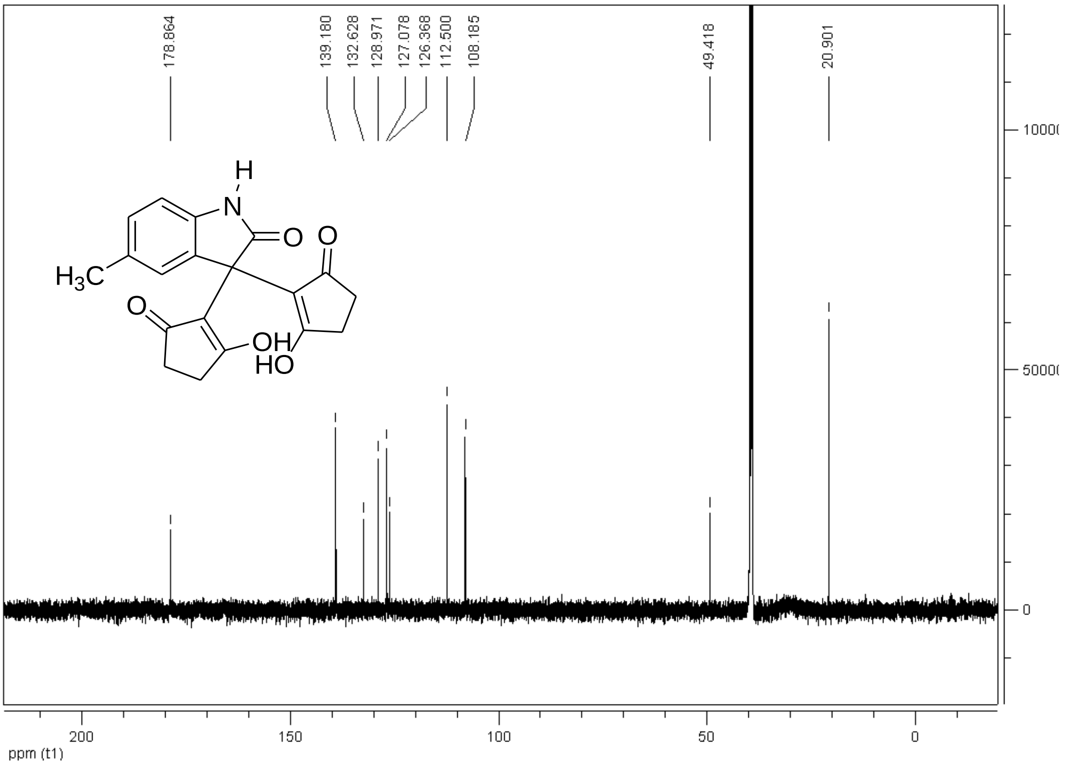

3c: white solid, 85%, mp 274–276 °C; ^{1}H NMR (600 MHz, DMSO-d_6) δ: 11.60 (brs, 2H, OH), 10.43 (s, 1H, NH), 7.21 (s, 1H, ArH), 7.07 (d, *J* = 8.4 Hz, 1H, ArH), 6.68 (d, *J* = 8.4 Hz, 1H, ArH), 2.30 (brs, 8H, CH_2); ^{13}C NMR (150 MHz, DMSO-d_6) δ: 177.7, 172.0, 140.7, 135.2, 126.5, 124.8, 124.6, 124.5, 112.1, 109.7, 49.2, 30.2, 30.1, 30.0, 29.9, 29.8, 21.0; IR (KBr) υ: 3186, 2929, 1672, 1608, 1571, 1477, 1436, 1387, 1310, 1233, 1179, 1122, 1064, 888, 849, 816 cm^{-1}; MS (*m*/*z*): HRMS (ESI) Calcd. for $C_{18}H_{13}ClNO_5$ ([M-H]$^-$): 358.0488 Found: 358.0488.

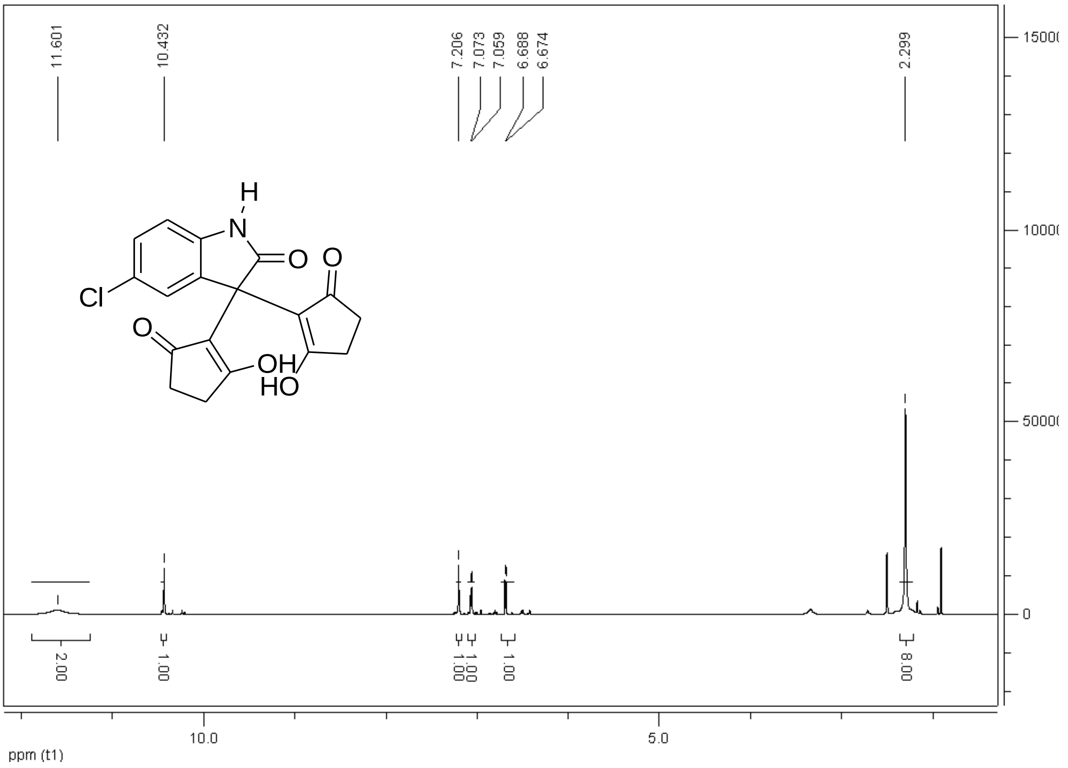

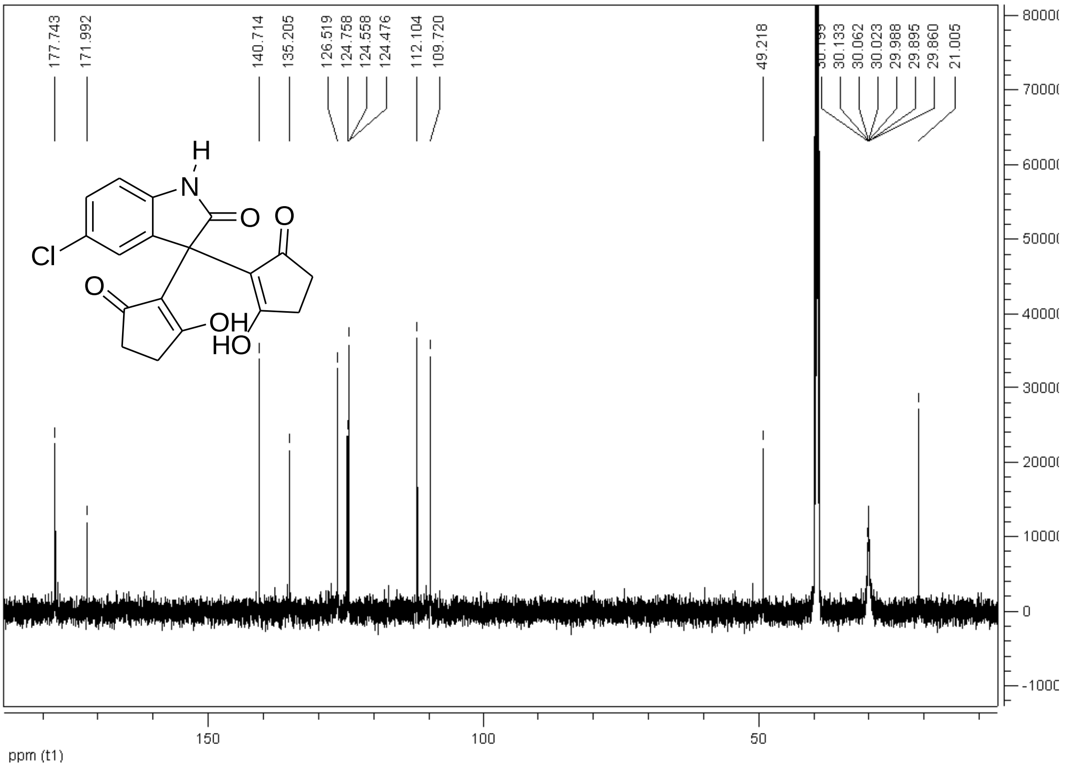

3d: white solid, 83%, mp 270–272 °C; ^{1}H NMR (600 MHz, DMSO-*d*$_6$) δ: 11.52 (brs, 2H, OH), 10.40 (s, 1H, NH), 7.02 (d, *J* = 9.0 Hz, 1H, ArH), 6.85 (d, *J* = 9.0 Hz, 1H, ArH), 6.67–6.65 (m, 1H, ArH), 2.30 (brs, 8H, CH_2); ^{13}C NMR (150 MHz, DMSO-*d*$_6$) δ: 178.4, 172.0, 158.2, 156.7, 137.9, 134.7, 134.6, 113.0, 112.9, 112.7, 112.0, 108.9, 108.8, 49.6, 30.5, 30.4, 30.3, 30.1, 30.0, 29.7, 21.0; IR (KBr) υ: 3194, 2932, 1675, 1608, 1574, 1488, 1434, 1389, 1363, 1312, 1195, 1069, 884, 850, 813 cm^{-1}; MS (*m*/*z*): HRMS (ESI) Calcd. for $C_{18}H_{13}FNO_5$ ([M-H]$^-$): 342.0782 Found: 342.0783.

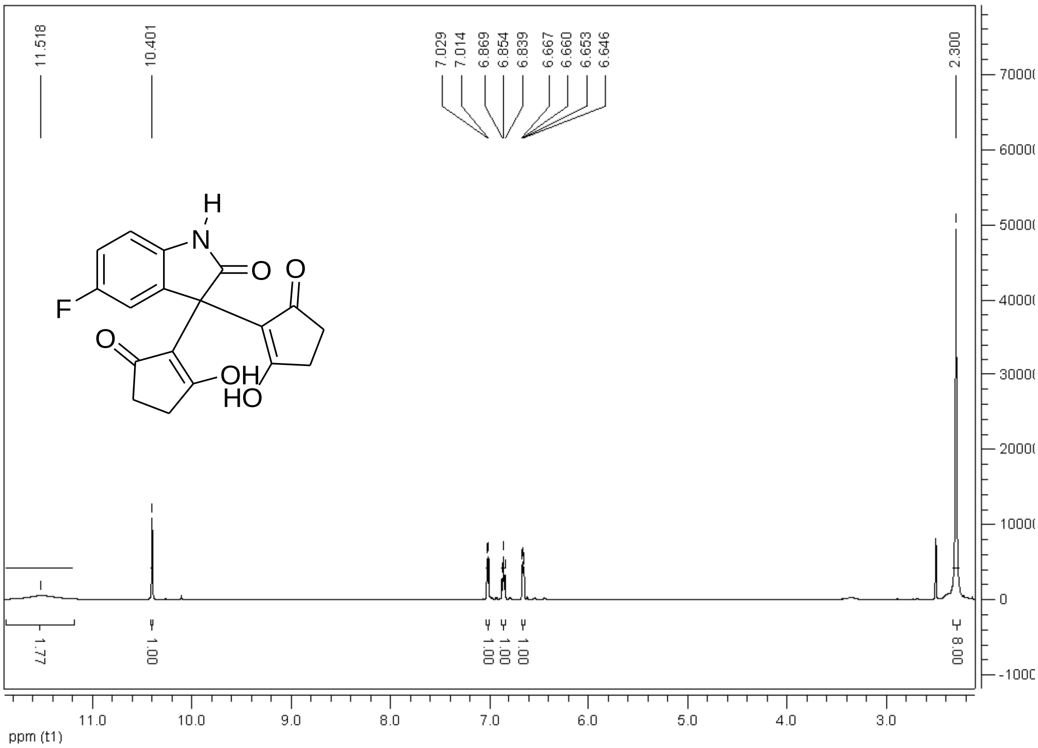

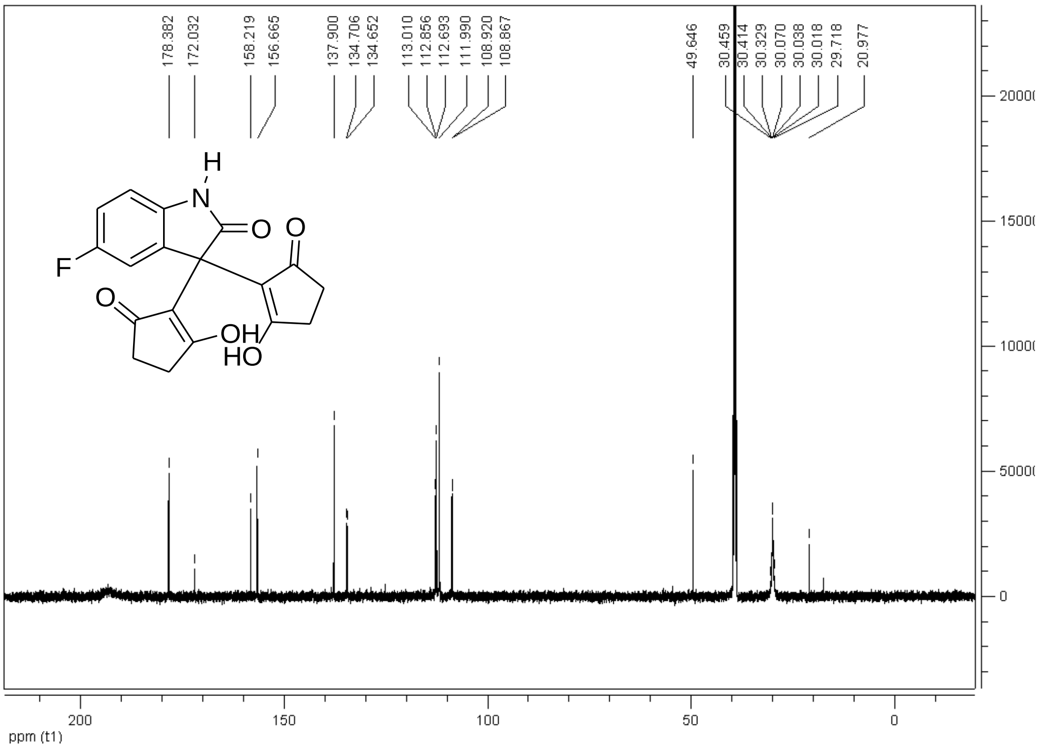

Supplement: File 1 — Spectroscopic and analytical data. [file Beilstein_J_Org_Chem-09-08-s001.pdf]
